# Intrinsically disordered proteins in the nucleus of human cells

**DOI:** 10.1016/j.bbrep.2015.03.003

**Published:** 2015-03-24

**Authors:** Telma Frege, Vladimir N. Uversky

**Affiliations:** a Department of Molecular Medicine, Morsani College of Medicine, University of South Florida, Tampa, FL 33612, USA; b GenomeNext LLC, 175 South 3rd Street, Suite 200, Columbus OH 43215, USA; c USF Health Byrd Alzheimer׳s Research Institute, Morsani College of Medicine, University of South Florida, Tampa, FL 33612, USA; d Department of Biology, Faculty of Science, King Abdulaziz University, P.O. Box 80203, Jeddah 21589, Saudi Arabia; e Institute for Biological Instrumentation, Russian Academy of Sciences, 142290 Pushchino, Moscow Region, Russia; f Laboratory of Structural Dynamics, Stability and Folding of Proteins, Institute of Cytology, Russian Academy of Sciences, St. Petersburg, Russia

**Keywords:** Intrinsically disordered proteins, Cell nucleus, *Homo sapiens*, Protein function, Nuclear proteins, Nuclear compartments

## Abstract

Intrinsically disordered proteins are known to perform a variety of important functions such as macromolecular recognition, promiscuous binding, and signaling. They are crucial players in various cellular pathway and processes, where they often have key regulatory roles. Among vital cellular processes intimately linked to the intrinsically disordered proteins is transcription, an intricate biological performance predominantly developing inside the cell nucleus. With this work, we gathered information about proteins that exist in various compartments and sub-nuclear bodies of the nucleus of the human cells, with the goal of identifying which ones are highly disordered and which functions are ascribed to the disordered nuclear proteins.

## 1 Introduction

Being the first discovered cellular organelle, the nucleus, this membrane enclosed organelle found in eukaryotic cells, was described for the first time by the early microscopist Antonie van Leeuwenhoek (1632–1723). The nucleus is a key component of the eukaryotic cell since it is the “container” of its genetic information that serves as the “control center” of the cell, which is responsible for the storage of genetic information and coordination of gene expression [1], [2], [3]. The number of nuclei within a cell varies between the species from one and four, with one being the most common case. This organelle generally occupies about 6% of the total size of the cell. Among the most important functions ascribed to the nucleus are: storage of hereditary material (in chromosomes and genes); storage of proteins and RNA (specifically in the nucleolus); exchange of hereditary molecules (DNA and RNA); and production of ribosomes. The nucleus is a dynamic organelle, whose morphology (size and shape) is tightly regulated and is noticeably changed during the cell cycle [4]. There is a correlation between altered nuclear morphology and development of some diseases, e.g., cancer [4].

**Fig. 1:**
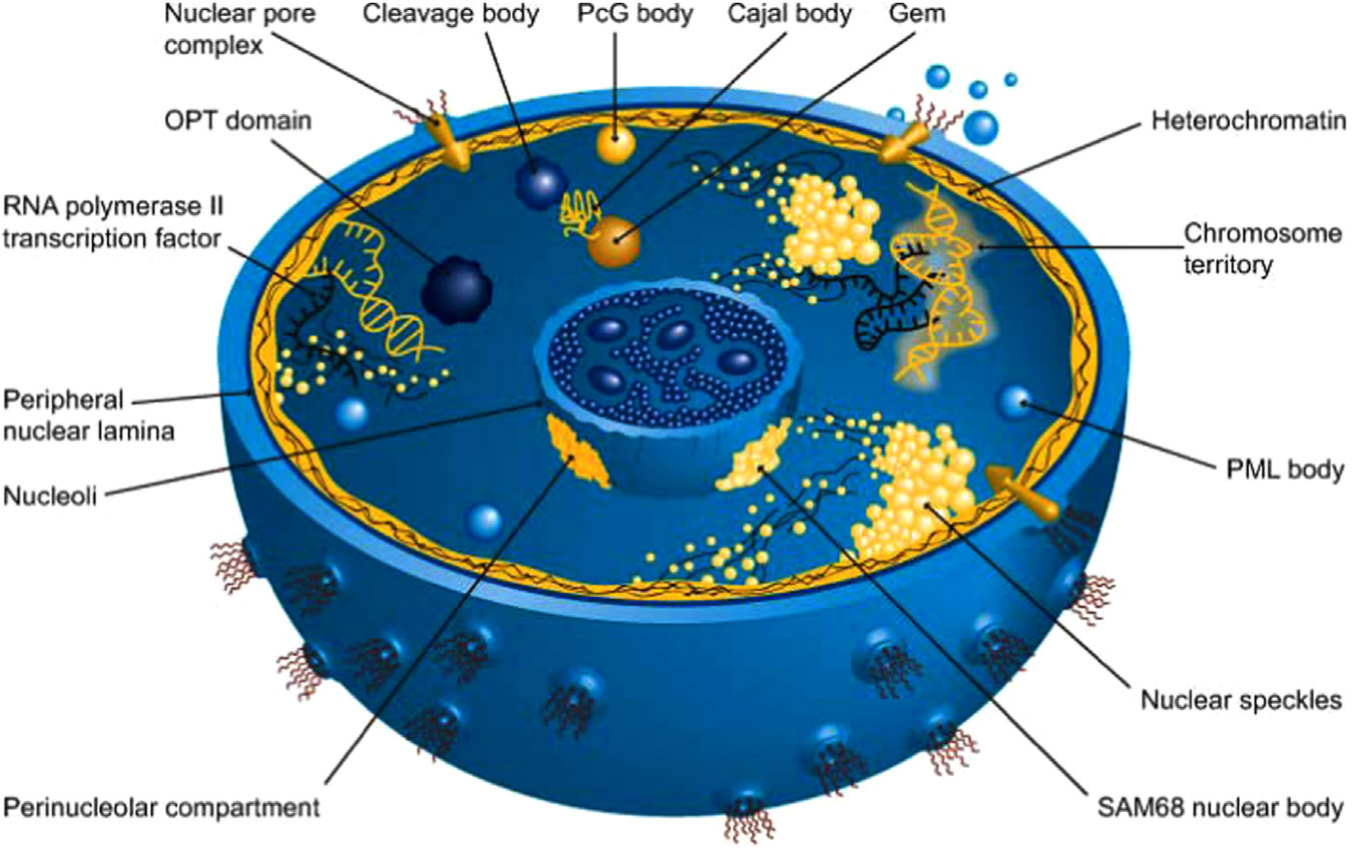
Sub-nuclear compartments. Reproduced with the permission from [211].

The cell nucleus is not a homogeneous entity, but contains several structures or compartments and sub-nuclear bodies [5]. Contrary to other components of the cell, most of these compartments are highly dynamic (do not exist all the time but only during certain stages of the cell, when those compartments are needed), and many of them are membrane-less, being formed via recruitment of proteins, RNA and DNA. Fig. 1 represents a schematic model of this membrane-enclosed organelle and shows that nucleus contains numerous nuclear domains or subnuclear organelles, such as nuclear pores, chromatin, nucleolus, PcG bodies (subnuclear organelles containing polycomb group proteins), Cajal bodies, promyelocytic leukemia (PML) nuclear bodies, Oct1/PTF/transcription (OPT) domains, nuclear speckles, nuclear gems (Gemini of coiled bodies), cleavage bodies, SAM68 nuclear bodies, perinuclear compartment, and several others [6]. Despite being different morphologically and functionally, all the aforementioned nuclear domains have some common features, e.g., all of them contain various types of RNA (or, in some cases, DNA) and different proteins.

Four levels of structural organization are known for a foldable ordered protein. Here, primary structure refers to the product of transcription, the protein amino acid sequence. Secondary structure corresponds to the 3D form of specific local segments, such as α-helix or β-strand. Tertiary structure represents the spatial conglomeration of secondary structure elements into a 3D super-structure. Tertiary structure is the highest form of the structural organization of a single-chain protein, whereas a multi-chain protein has quaternary structure that represents a more complex structural level constituting the assembly of tertiary structures.

Although for a long time it has been assumed that the presence of unique structure is a crucial prerequisite for protein to be functional, recent studied revealed that many biologically active proteins are characterized by a lack of tertiary structure. The discovery of these intrinsically disordered proteins (IDPs) and hybrid proteins consisting ordered domains and intrinsically disordered protein regions (IDPRs) challenged the protein structure paradigm stating that a protein must have a defined 3D-structure in order to perform a function [7], [8], [9], [10], [11], [12], [13]. Studies of different genomes suggested that IDPs are very abundant in nature and that proteins from eukaryotes have more intrinsic disorder than proteins of bacteria or archaea (up to 42% of all proteins in humans) [10], [14], [15], [16], [17], [18], [19], [20].

Besides being very common in all analyzed proteomes, IDPs/IDPRs were shown to possess unique functional repertoire (being commonly involved in regulation, signaling and control pathways [21], [22], [23]), which is complementary to catalytic and transport functions of ordered proteins [24], [25], [26], [27].

Since among the disorder-specific functions are DNA- and RNA-interactions, the goal of this work was to study the nuclear proteins of the human cell, to evaluate their level of disorder and to see if there is any sort of connection between intrinsic disorder and the functional roles these proteins play in the cell. The study is challenged by the fact that the information on components of the nucleus of human cells is scarce. Little information is currently available about the highly dynamic environment of this important cellular compartment, its organelles and interactions between them and the roles each such organelle plays. Furthermore, the molecular mechanisms defining the ability of these nuclear domains to maintain their specific structures in the absence of membranes also remain mostly unknown [28]. Studies of the proteins containing in each of these organelles (also called sub-nuclear domains or compartments) are sparse. Therefore, the overall goals of this project were: to find the proteins located in the human nucleus; to analyze distribution of these proteins within the sub-nuclear compartments; to analyze functions and structures of these proteins; to evaluate the intrinsic disorder propensities of these proteins; to look at the roles of intrinsic disorder in function and regulation of nuclear proteins; to map the distribution of disorder propensity within the nuclear compartments; and to study the relationship – if any – between the dynamic organelles within the cell nucleus and the level of disorder in the proteins that make them up.

## 2 Materials and methods

To solve this problem, a series of existing tools and data sources were used, and with them a pipeline was built to collect and process the information needed. Fig. S1 represents the resulting pipeline that had three major processing points, data collection, data processing, and data analysis.

### 2.1 Data collection

This stage had two steps. On the first step, the Nuclear Protein Database [29] was used to identify the proteins located inside the nucleus of eukaryotic cells. A total of 795 proteins were identified. On the second step, each protein was checked against UniProt to narrow the dataset to proteins that were curated and of human origin. The request for a protein to be curated provided us with the possibility to work only with proteins that have been reviewed and have reliable information. The protocol of protein curation in UniProt is shown in Fig. S1. The proteins of interest should be of human origin to filter out other species and focus on *Homo sapiens* only. The output of this stage was 185 proteins, collected in text files (FASTA sequences) and XML files. Each XML file contained all the details that UniProt provided for a given protein including all the names used for a protein, codes, cell location(s), functions, processes, sequence, etc. The distribution of these proteins in the different nucleus compartments is shown in Table 1. It is worth mentioning that the nucleus of the human cell has more proteins, but at the time this project started, the Nuclear Protein Database (http://npd.hgu.mrc.ac.uk/) [29] contained only 185 human curated proteins.

**Table 1:** Distribution of the 185 curated human proteins among the sub-nuclear compartments and the average disorder contents of these proteins.

**Compartment**	**Number of proteins**	**Average disorder (%)**
Cleavage body	2	83
OPT domain	2	78
SAM68 nuclear body	1	72
Nuclear speckles	12	65
Chromatin	1	60
PcG body	8	59
Nuclear pore complex	15	55
Cajal body	6	54
Perinuclear compartment	1	52
Heterochromatin	43	51
Nucleolus	86	44
PML body	1	41
RNA polymerase II	6	27
Gem	3	25

### 2.2 Data processing

On the second stage, which also had two steps, the 185 proteins were analyzed with a set of disorder predictors. There were several choices when it came to choose one, depending on the way the disorder is predicted. Because of their reputations and the detailed information they provide, the binary classifier CH-CDF plot and the PONDR-FIT^®^ metapredictor [30] were chosen for this project.

#### 2.2.1 PONDR-FIT^®^ processing

PONDR-FIT^®^ is a protein disorder meta-predictor (combines several methods to predict the level of disorder of a given sequence). This tool has proven to be moderately more accurate than many other disorder predicting tools [30]. The input for PONDR-FIT^®^ is the FASTA sequences of the proteins. The tool returns a series of files for each protein, such as a META file that contains detailed prediction information for the protein to the amino acid level; VSL2 file that contains the sequence of residues and two columns with the results of disorder prediction for each residue (a number between 0 and 1) and a ‘flag’ field indicating if the residue is considered to be disordered (‘D’ if the prediction is greater than 0.5, ‘.’ otherwise). Furthermore, the VSL2 output file contains, the header representing a summary of the regions that are predicted as disordered; VLXT file containing the sequence of residues and the POND VLXT disorder score for each residue (a number between 0 and 1).

In addition to these files with raw data, the in-house version of PONDR-FIT^®^ returns a unique file with a summary of the findings for all the proteins processed. The columns of this file are:

• 1st column: name
• 2nd column: length of the protein
• 3rd and 4th: R%_meta and No – fraction of disordered residues and number of disordered segments in the protein predicted by PONDR-FIT;
• 5th and 6th: R%_VLXT and No – fraction of disordered residues and number of disordered segments in the protein predicted by PONDR-VLXT;
• 7th and 8th: R%_VL3 and No – fraction of disordered residues and number of disordered segments in the protein predicted by PONDR-VL3;
• 9th and 10th: R%_VSL2B and NO – fraction of disordered residues and number of disordered segments in the protein predicted by PONDR-VSL2B;
• 11th and 12th: R%_IUPred and NO – fraction of disordered residues and number of disordered segments in the protein predicted by IUPred;
• 13th and 14th: R%_FD and NO – fraction of disordered residues and number of disordered segments in the protein predicted by FoldIndex;
• 15th and 16th: R%_Top and NO – fraction of disordered residues and number of disordered segments in the protein predicted by TopIDP;
• 18th: CH_charge – averaged net charges
• 19th: CH_hydro – averaged hydrophobicity
• 20th: CH_dist – spatial distance on CH plot
• 21st: CH_dist2 – vertical distance on CH plot
• 22nd and 23th: N_CDFx and dCDFx – points above CDF boundary and distance from CDF boundary calculated by PONDR-VLXT prediction;
• 24th and 25th: N_CDFs and dCDFs – points above CDF boundary and distance from CDF boundary calculated by PONDR-VSL2B prediction;
• 26th and 27th: N_CDF3 and dCDF3 – points above CDF boundary and distance from CDF boundary calculated by PONDR-VL3 prediction;
• 28th and 29th: N_CDFi and dCDFi – points above CDF boundary and distance from CDF boundary calculated by IUpred prediction;
• 30th and 31st: N_CDFf and dCDFf - points above CDF boundary and distance from CDF boundary calculated by FoldIndex prediction;
• 32nd and 33th: N_CDFt and dCDFt – points above CDF boundary and distance from CDF boundary calculated by TopIDP prediction;

On the second step of this stage, all the information collected up to this moment in various files was loaded in a MySQL database using PHP scripts. There was no particular reason to choose both tools (MySQL, PHP) aside from the fact that the development and execution were quick, both are open source tools, and the extensive experience of the author of the pipeline with both technologies.

Table 2 represents the PHP scripts that were designed to complete the following tasks:

1. Load and read of all the files of a given folder and a file type (meta, vls2, etc.).
2. Parse of information, breaking down each row and columns, cutting the white spaces and transforming the strings into numbers when necessary.
3. Insert of the resulting values into a table in a MySQL Database. Each file had a table related and additional tables were built to store the information shared across the files, like the protein name and compartment.
4. Pull the information of each protein from UniProt in XML format.
5. Read the information of each protein and obtain its full name and list of functions.

**Table 2:** The PHP scripts written in this study.

**PHP script name**	**Function**
**compartments.php**	Relate the compartment with the protein code, and store that relationship in the “COMPARTMENT” table.
**longregions.php**	Once the data from the vsl2 files is loaded, this script reads the vsl2_files table and calculates the long disordered regions (regions with 30 or more consecutive disordered residues).
	This script then stores the results in the table “LONG_DISORDERED_REGIONS”, saving: the protein code, the position where the region starts and the length of the region.
**meta.php**	Load and process all the .meta files. The results are stored in the table “META_FILES”.
**sum.php**	Load and process all the pondrfit summary files. The results are stored in the table “PONDFIT_SUM”.
**vlxt.php**	Load and process all the .vlxt files. The results are stored in the table “VLXT_FILES”.
**vsl2.php**	Load and process all the .vsl2 files. The results are stored in the table “VSL2_FILES”.
**pullXmlUniprot.php**	Look for each protein at Uniprot.org and pull its XML.
**parseXmlUniprot.php**	Open each of the files retrieved by pullXmlUniprot.php, and look for the nodes in the XMLs that list the protein׳s biological processes and functions, and store them in an Excel file (that later will be loaded to the Database with the help of a EDI).

Having all the data loaded in a Relational Database facilitated the structuring of the information and the easy querying and filtering. The results from the PHP scripts were stored in a Database created in MySQL. The resulting E-R (entity-relationship) diagram is shown in Fig. S2, whereas Fig. S3 shows view created from different tables.

#### 2.2.2 Binary classification based on the CH-CDF analysis

In CD-CDF plot, coordinates of each spot are calculated as a distance of the corresponding protein in the CH-plot (charge-hydropathy plot) [9], [17] from the boundary (*Y*-coordinate) and an average distance of the respective cumulative distribution function (CDF) curve [17] from the CDF boundary (*X*-coordinate) [31], [32], [33]. The primary difference between these two binary predictors (i.e., predictors which evaluate the predisposition of a given protein to be ordered or disordered as a whole) is that CH-plot is a linear classifier that takes into account only two parameters of the particular sequence (charge and hydropathy), whereas CDF analysis is dependent on the output of the PONDR^®^ predictor, a nonlinear classifier, which was trained to distinguish order and disorder based on a significantly larger feature space. According to these methodological differences, CH-plot analysis is predisposed to discriminate proteins with substantial amount of extended disorder (random coils and pre-“molten globules”) from proteins with compact conformations (“molten globule”-like and rigid well-structured proteins). On the other hand, PONDR-based CDF analysis may discriminate all disordered conformations, including molten globules, from rigid well-folded proteins. Therefore, this discrepancy in the disorder prediction by CDF and CH-plot provides a computational tool to discriminate proteins with extended disorder from molten globule-like compact IDPs or hybrid proteins containing ordered domains and IDPRs. Positive and negative *Y* values in CH-CDF plot correspond to proteins predicted within CH-plot analysis to be natively unfolded or compact, respectively. On the contrary, positive and negative *X* values are attributed to proteins predicted within CDF analysis to be ordered or intrinsically disordered, respectively. Thus, the resultant quadrants of CDF-CH phase space correspond to the following expectations: Q1, proteins predicted to be disordered by CH-plots, but ordered by CDFs; Q2, ordered proteins; Q3, proteins predicted to be disordered by CDFs, but compact by CH-plots (i.e., putative molten globules or hybrid proteins with ordered domains and IDPRs); Q4, proteins predicted to be disordered by both methods.

#### 2.2.3 Disorder analysis with MobiDB

Disorder evaluations for human nuclear proteins were further enhanced by utilizing the outputs of the MobiDB database (http://mobidb.bio.unipd.it/), [34], [35] that generates consensus disorder scores based on the outputs of ten disorder predictors, such as ESpritz in its two flavors, [36] IUPred in its two flavors, [37] DisEMBL in two of its flavors, [38] GlobPlot, [39] PONDR^®^ VSL2B, [40], [41] and JRONN [42].

#### 2.2.4 Focused look on some human nuclear proteins

For several human nuclear proteins, disorder evaluations together with important disorder-related functional information was retrieved from D^2^P^2^ database (http://d2p2.pro/) [19]. D^2^P^2^ is a database of predicted disorder that represents a community resource for pre-computed disorder predictions on a large library of proteins from completely sequenced genomes [19]. D^2^P^2^ database uses outputs of PONDR^®^ VLXT [43], IUPred [37], PONDR^®^ VSL2B [40], [41], PrDOS [44], ESpritz [36] and PV2 [19]. This database is further enhanced by information on the curated cites of various posttranslational modifications and on the location of predicted disorder-based potential binding sites.

#### 2.2.5 Finding potential disorder-based binding sites

Potential binding sites in disordered regions can be identified by the ANCHOR algorithm [45], [46]. This approach relies on the pairwise energy estimation approach developed for the general disorder prediction method IUPred, [37], [47] being based on the hypothesis that long regions of disorder contain localized potential binding sites that cannot form enough favorable intrachain interactions to fold on their own, but are likely to gain stabilizing energy by interacting with a globular protein partner [45], [46]. Regions of a protein suggested by the ANCHOR algorithm to have significant potential to be binding sites are the ANCHOR-indicated binding site (AIBS).

### 2.3 Data analysis

The last step was about analyzing the data loaded in MySQL using Structured Query Language (SQL). The goals of these stages were to:

• Group the proteins by their percentage of disorder.
• Find the compartments of the nucleus that have a high number of disordered proteins.
• Find functions and processes of highly disordered proteins.
• See if some function(s) or process(es) is(are) common among these groups of proteins, suggesting that their lack of structure has a direct impact in the function.
• Find functions and processes of highly structured proteins.
• Compare these proteins׳ functions and processes with those found among the disordered ones.

To accomplish this, the following were used:

• SQL queries in MySQL.
• Views built in MySQL.
• Graphs created in Excel.

## 3 Results

**Fig. 2:**
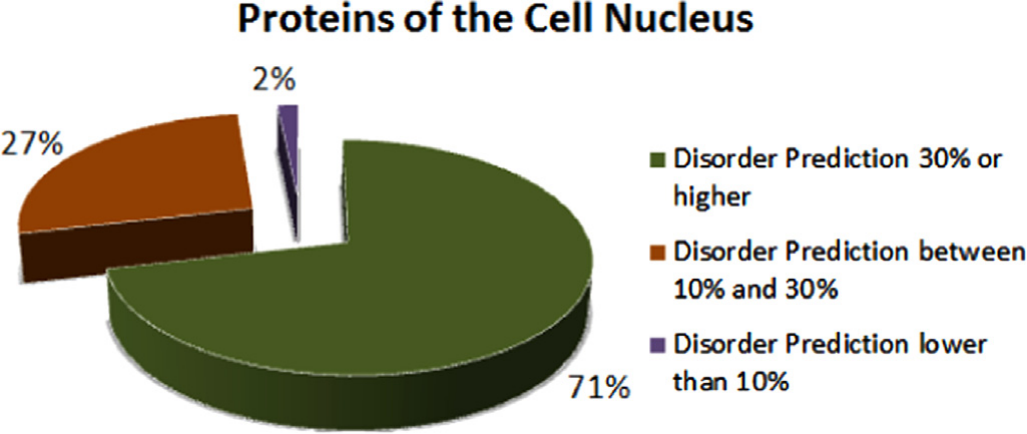
Peculiarities of disorder distribution in human nuclear proteins.

The overall disorder contents of various nuclear domains are listed in Table 1, whereas Table S1 represents details of disorder prediction for each of 185 human nuclear proteins analyzed in this study. These data clearly show that according to the accepted classification where two arbitrary cutoffs for the levels of intrinsic disorder are used to classify proteins as highly ordered (IDP score<10%), moderately disordered (10%≤IDP score<30%) and highly disordered (IDP score≥30%), [48] the majority of cellular suborganelles are strongly disordered. This idea is further illustrated by Fig. 2 representing a pie-chart that shows the distribution of human nuclear proteins among these three disorder categories.

**Fig. 3:**
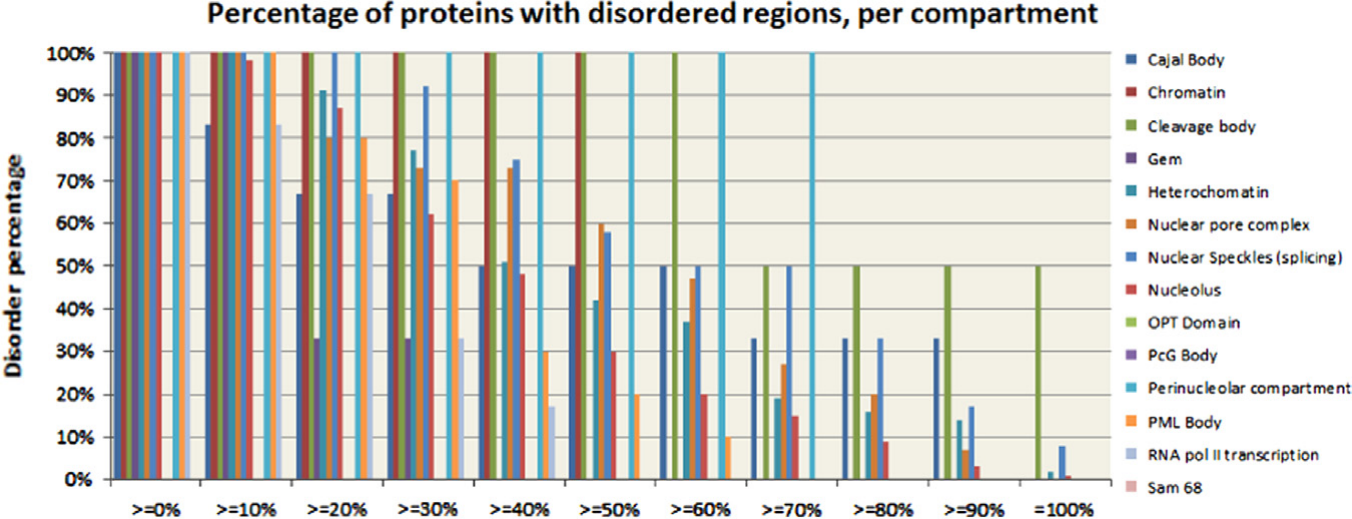
Detailed representation of the disorder distribution in various nuclear compartments. This bar graph shows how many proteins in each compartment have disordered regions accounting for ≥0%, ≥10%, ≥20%, …, ≥80%, ≥90%, and 100% of their residues.

Fig. 3 provides the peculiarities of the disorder distribution per nuclear compartment. Here, plot shows how many proteins in a given compartment have that percentage of disorder or greater. For example, heterochromatin has 43 proteins with 10% or more disorder, representing 100% of its proteins. The same compartment has 39 proteins (or 91%) with 20% or more disorder, and 33 proteins (77%) with 30% or more disorder, and so on. Therefore, Fig. 3 gives a visual representation of the peculiarities of intrinsic disorder distribution within the nuclear compartments analyzed in this study and shows that a significant faction of proteins in various nuclear compartments correspond to proteins with high intrinsic disorder contents (i.e., proteins containing at least 50% of disordered residues).

**Fig. 4:**
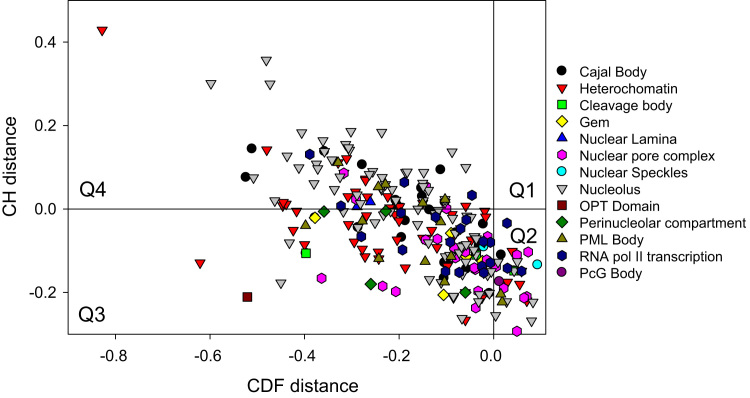
Evaluating intrinsic disorder in human nuclear proteins by combined binary disorder classifiers, CH-plot [9] and CDF [9], [17], [33]. Here, the coordinates of each point were calculated as a distance of the corresponding protein in the CH-plot from the boundary (*Y*-coordinate) and an average distance of the respective CDF curve from the CDF boundary (*X*-coordinate). The four quadrants correspond to the following predictions: Q1, proteins predicted to be disordered by CH-plots, but ordered by CDFs; Q2, ordered proteins; Q3, proteins predicted to be disordered by CDFs, but compact by CH-plots (i.e., putative molten globules or hybrid proteins); Q4, proteins predicted to be disordered by both methods.

To get further insight into the nature of disorder in human nuclear proteins, the binary CH-CDF classifier was applied. Fig. 4 represents the results of this analysis and clearly shows the by their overall level of intrinsic disorder human nuclear proteins can be grouped into three classes according to their localization within the CH-CDF phase space. In fact, the vast majority of proteins in almost all nuclear suborganelles are expected to disordered as a whole and behave either as native coils or native pre-molten globules (i.e., predicted as disordered by CH and CDF), or potential native molten globules/hybrid proteins (predicted as disordered by CDF but as compact by CH-plot). Only ~12% of these proteins are expected to be ordered as whole, being predicted as ordered by both CH and CDF tools. There is no single protein in this set which would be predicted to be ordered by CDF and disordered by CH-plot analysis.

**Fig. 5:**
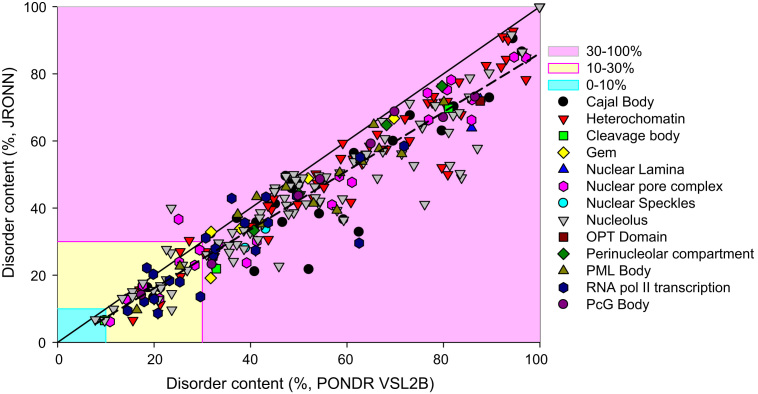
Abundance of intrinsic disorder in human nuclear proteins. JRONN vs. PONDR^®^ VSL2B plot representing the correlation between the disorder content evaluated by PONDR^®^ VSL2B (*x*-axis) [40], [41] and by JRONN (*y*-axis) [42]. Solid black line corresponds to the diagonal. Dashed line shows linear fit of all the data point to the equation (*R*^2^=0.90):.$DS_{JRONN}=–1.4_{\pm 1}+0.85_{\pm 0.02}\times DS_{PONDR}.$ Following the accepted practice, two arbitrary cutoffs for the levels of intrinsic disorder are used to classify proteins as highly ordered (IDP score<10% light blue field), moderately disordered (10%≤IDP score<30%, light yellow field) and highly disordered (IDP score≥30%, light pink field) [48].

The point that human nuclear proteins analyzed in this study are mostly disordered is further illustrated in Fig. 5 that compares the percentage of disorder evaluated for the different nuclear proteins by PONDR^®^ VSL2B and JRONN. Fig. 5 shows that the results of the disorder predictions obtained by these two computational tools are mostly agree with each other. The aforementioned classification of proteins as highly ordered (IDP score<10%), moderately disordered (10%≤IDP score<30%), and highly disordered (IDP score≥30%) [48] is visualized in this plot as light blue, light yellow, and light pink areas, respectively. Fig. 5 shows that according to this classification and based on the results of disorder evaluation by the two predictors, almost all human nuclear proteins are predicted as either moderately or highly disordered.

Often, intrinsically disordered protein regions (IDPRs) contain local regions with a strong tendency to become ordered. These regions might undergo coupled folding and binding resulting from their interaction with corresponding binding partners (e.g., for some early NMR studies see Refs. [49], [50], [51], [52], [53], [54]). Furthermore, predictions of local order within long disordered regions coincide with potential binding sites [55]. Therefore, molecular recognition features (MoRFs) can be found as short regions with increased order propensity and high α-helix-forming propensity that are located within the long disordered regions and undergo coupled binding and folding short regions [56], [57]. Systematic application of this computational tool to databases of genomics and functionally annotated proteins indicated that α-MoRFs are likely to be play important roles protein–protein interactions involved in signaling events [56]. Alternatively, disorder-based potential binding sites can be found using the ANCHOR algorithm [45], [46]. These ANCHOR-indicated binding sites (AIBSs) found as a result of the analysis of 185 human nuclear proteins are listed in Table S1. This analysis revealed that AIBS are present in all nuclear proteins which contain at least one AIBS. Furthermore, all but one nuclear protein were shown to contain multiple AIBSs. In fact, 2231 AIBSs were found in 185 human nuclear proteins, suggesting that, on average, each protein contained ~12 AIBS. Table S1 shows that only 28 proteins contain less than 5 AIBSs, and there are 23 proteins that have more than 20 AIBSs each. The largest number of AIBSs (97) is found within the antigen KI-67 (UniProt ID: P46013) located within the heterochromatin (see Table S1). There are four more proteins containing at least 40 AIBSs each: nuclear receptor corepressor 2 (N-CoR2, UniProt ID: Q9Y618; 55 AIBSs), breast cancer type 1 susceptibility protein (BRCA1, UniProt ID: P38398, 44 AIBSs), protein SON (also known as Bax antagonist selected in saccharomyces 1 (Bass1) or negative regulatory element-binding protein (NRE-binding protein), UniProt ID: P18583, 43 AIBSs), and transcriptional regulator ATRX (UniProt ID: P46100, 40 AIBSs). Furthermore, Table S1 shows that all nuclear compartments contained multi-AIBS proteins and that length of the AIBSs was ranging from 6 to 211 residues. The presence of more than one AIBS in a protein suggests that almost all nuclear proteins are involved either in the polyvalent interactions by using multiple binding sites to interact with one binding partner or in scaffolding-like interactions by using multiple binding sites to interact with multiple binding partners. The wide spread of lengths of identified AIBSs also suggests the presence of multiple disorder-based binding mechanisms (ranging from local folding-on-binding of short regions to wrapping around binding mode to global binding-induced folding of large regions). The high abundance of AIBS within human nuclear proteins suggests that these disorder-based features are commonly utilized by these proteins for their interactions with binding partners.

**Fig. 6:**
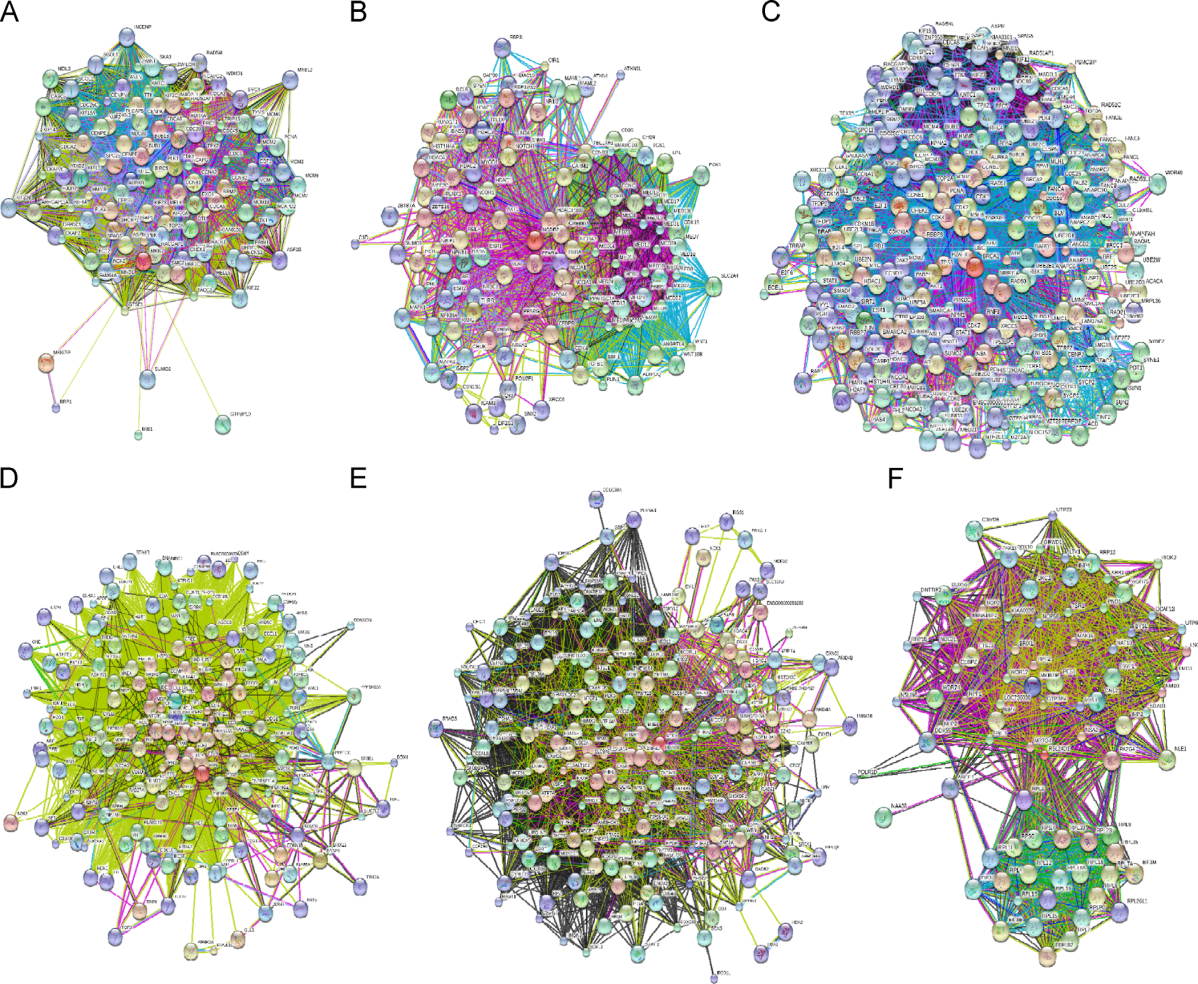
Analysis of the interactivity of the several human nuclear proteins by the STRING platform [58]. Analyzed proteins are: (A) KI-67 (UniProt ID: P46013), (B) N-CoR2 (UniProt ID: Q9Y618), (C) BRCA1 (UniProt ID: P38398), (D). SON (UniProt ID: P18583), (E) ATRX (UniProt ID: P46100), and (F) RLP24 (UniProt ID: Q9UHA3). STRING produces the network of predicted associations for a particular group of proteins. The network nodes are proteins, whereas the edges represent the predicted or known functional associations. An edge is drawn with up to 7 differently colored lines that represent the existence of the seven types of evidence used in predicting the associations. A red line indicates the presence of fusion evidence; a green line – neighborhood evidence; a blue line – co-occurrence evidence; a purple line – experimental evidence; a yellow line – text mining evidence; a light blue line – database evidence; a black line – co-expression evidence [58].

Fig. 6 represents illustrative examples of interaction networks for five nuclear proteins, which, according to the ANCHOR analysis, contained the largest number of disorder-based binding sites. These proteins are: KI-67 (UniProt ID: P46013, Fig. 6A), N-CoR2 (UniProt ID: Q9Y618, Fig. 6B), BRCA1 (UniProt ID: P38398, Fig. 6C), SON (UniProt ID: P18583, Fig. 6D), and ATRX (UniProt ID: P46100, Fig. 6E). The interactivity of these human proteins was evaluated by the online database resource STRING (Search Tool for the Retrieval of Interacting Genes) that provides both experimental and predicted information on interactions of a protein of interest [58]. STRING produces the network of predicted associations for a particular group of proteins. The network nodes are proteins, whereas the edges represent the predicted or known functional associations. An edge is drawn with up to 7 differently colored lines that represent the existence of the seven types of evidence used in predicting the associations. A red line indicates the presence of fusion evidence; a green line – neighborhood evidence; a blue line – co-occurrence evidence; a purple line – experimental evidence; a yellow line – text mining evidence; a light blue line – database evidence; a black line – co-expression evidence [58]. Fig. 5 clearly shows that in agreement with the high numbers of predicted potential disorder-based interaction sites in these five proteins, all of them serve as hubs of well-developed and exhaustive interaction networks. For comparison, Fig. 6F represents the results of STRING analysis of the probable ribosome biogenesis protein RLP24 (UniProt ID: Q9UHA3), which is a human nuclear protein with just one predicted AIBS. This protein too is involved in numerous interactions.

High binding promiscuity and high levels of connectivity predicted for these proteins indicates that these human nuclear proteins, which are also predicted to contain substantial amount of intrinsic disorder, belong to the family of disordered hub proteins, which are multitasking proteins found in protein–protein interaction networks (PPI), where these promiscuous binders have multiple links. Depending on timing of their interactions within the PPI networks, hubs are classified as “party hubs” if they are involved in multiple simultaneous interactions, whereas hubs with multiple sequential interactions are considered as “date hubs” [59]. Obviously, the biological role of the “date hubs” is to connect biological modules to each other [60], whereas “party hubs” have scaffolding roles by enabling the assembly of functional modules [59]. Binding promiscuity of hub proteins is believed to rely on intrinsic disorder [23], [61], [62], [63], [64], [65], that provides a molecular mechanism for one-to-many and many-to-one PPIs [7].

The next step was to identify the common functions performed by the proteins with the highest and lowest levels of disorder. The question to answer with this exercise was: “is there a relationship between the structure – or lack thereof – of these proteins and their functions?” Interestingly enough, several shared functions were identified for both disorder categories. Results of this analysis are shown in Table 3, Table 4, Table 5 which list functions and processed shared by seven or more highly disordered proteins and three or more moderately disordered proteins. There is a higher number of functions assigned to highly disordered proteins (Table 4) compared to the functions found among moderately disordered proteins (Table 5). This indicates that most of the functions in the nucleus of the cell (which are critical to the function and survival of the entire cell) depend on highly disordered proteins.

**Table 3:** Functions shared by highly disordered proteins.

**Type**	**Description**	**Count**	**% avg disorder**
PROCESS	Negative regulation of apoptotic process	7	77
PROCESS	Small molecule metabolic process	9	72
PROCESS	Mitotic nuclear envelope disassembly	8	72
PROCESS	Regulation of glucose transport	8	72
PROCESS	Transmembrane transport	8	72
PROCESS	Carbohydrate metabolic process	8	72
PROCESS	Glucose transport	8	72
PROCESS	Hexose transport	8	72
PROCESS	Cytokine-mediated signaling pathway	9	68
PROCESS	Mitotic cell cycle	15	66
PROCESS	Negative regulation of transcription from RNA polymerase II promoter	16	65
PROCESS	mRNA transport	9	64
PROCESS	mRNA processing	11	63
PROCESS	Negative regulation of transcription, DNA-templated	21	62
PROCESS	Transcription from RNA polymerase II promoter	11	62
PROCESS	Positive regulation of transcription from RNA polymerase II promoter	18	61
PROCESS	Transcription, DNA-templated	35	61
PROCESS	Viral process	17	60
PROCESS	Gene expression	18	60
PROCESS	RNA splicing	16	60
PROCESS	mRNA metabolic process	7	58
PROCESS	Positive regulation of transcription, DNA-templated	14	57
PROCESS	mRNA splicing, via spliceosome	11	56
PROCESS	Negative regulation of cell proliferation	7	56
PROCESS	RNA metabolic process	9	53
PROCESS	Regulation of transcription, DNA-templated	17	53
PROCESS	Negative regulation of cell growth	7	52
PROCESS	Cellular response to DNA damage stimulus	10	51
PROCESS	DNA repair	11	51
PROCESS	Cellular protein metabolic process	7	47
FUNCTION	Sequence-specific DNA binding transcription factor activity	11	65
FUNCTION	Chromatin binding	22	65
FUNCTION	DNA binding	33	63
FUNCTION	Transcription corepressor activity	10	61
FUNCTION	Poly(A) RNA binding	48	61
FUNCTION	Sequence-specific DNA binding	8	61
FUNCTION	Protein binding	91	59
FUNCTION	Enzyme binding	7	59
FUNCTION	Nucleotide binding	16	58
FUNCTION	Zinc ion binding	18	55
FUNCTION	Identical protein binding	11	55
FUNCTION	RNA binding	24	53
FUNCTION	Transcription coactivator activity	7	50
FUNCTION	ATP binding	17	50
FUNCTION	Metal ion binding	7	41

**Table 4:** Functions shared by moderately disordered proteins.

**Type**	**Description**	**Count**	**% avg disorder**
FUNCTION	Metal ion binding	3	15
FUNCTION	ATP binding	7	13
FUNCTION	ATP-dependent RNA helicase activity	3	13
FUNCTION	Protein binding	14	13
FUNCTION	RNA helicase activity	3	13
FUNCTION	DNA binding	7	12
FUNCTION	Poly(A) RNA binding	8	12
PROCESS	Protein transport	3	16
PROCESS	mRNA processing	3	14
PROCESS	Negative regulation of transcription from RNA polymerase II promoter	4	14
PROCESS	ATP catabolic process	3	13
PROCESS	DNA repair	4	12
PROCESS	Gene expression	5	12
PROCESS	Positive regulation of transcription from RNA polymerase II promoter	3	12
PROCESS	Innate immune response	4	11

**Table 5:** Functions found in common between highly and moderately disordered nuclear proteins.

**Type**	**Description**
FUNCTION	ATP binding
FUNCTION	DNA binding
FUNCTION	Metal ion binding
FUNCTION	Poly(A) RNA binding
FUNCTION	Protein binding
PROCESS	ATP catabolic process
PROCESS	DNA repair
PROCESS	Gene expression
PROCESS	Innate immune response
PROCESS	mRNA processing
PROCESS	Negative regulation of transcription from RNA polymerase II promoter
PROCESS	Positive regulation of transcription from RNA polymerase II promoter
PROCESS	Protein transport

## 4 Discussion

The space between the nuclear envelope and the nucleolus is filled by a transparent, semi-solid, granular substance known as nucleoplasm, nuclear sap or karyolymph. It is composed mainly of nucleoproteins but also contains organic and inorganic molecules, such as nucleic acids (DNA and RNA), minerals, and enzymes needed for the synthesis of DNA, RNA and ribosomal subunits. These and other nuclear components are not uniformly distributed within the nucleoplasm, and nucleus contains several temporary membrane-less organelles (nuclear domains or nuclear suborganelles, or subnuclear organelles, or subnuclear bodies), such as nucleolus, chromatin, PcG bodies, Cajal bodies, PML nuclear bodies, OPT domains, nuclear speckles, nuclear gems, cleavage bodies, SAM68 nuclear bodies, perinuclear compartment, and several others (see Fig. 1) [6]. Sections below represent several subnuclear bodies and consider some prominent examples of the experimentally validated intrinsically disordered proteins from these nuclear suborganelles.

### 4.1 Disorder in cleavage body (average disorder score 83%)

Cleavage bodies have diameters of 0.3–1 μm and range in number from 1 to 4 per nucleus [66]. These suborganelles got their name from the fact that they contains the cleavage stimulation factor (CstF), and the cleavage and polyadenylation specificity factor (CPSF), both of which are necessary for 3′-terminal processing of polyadenylated mRNAs [66]. Besides these cleavage factors cleavage bodies are known to contain transcription factors TFIIE and TFIIF [67]. One of proteins colocalized with cleavage bodies is prothymosin *α* [68], structural properties which were characterized by a multitude of biophysical techniques to show that it is a typical highly extended IDP with almost complete lack of residual secondary structure [69], [70], [71].

### 4.2 Disorder in OPT domains (average disorder score 80%)

Oct1/PTF/transcription (OPT) domains are active domains that contain nascent transcripts, rich in transcription factors (PTF, Oct1, TBF, and Sp1), and includes RNA polymerase II. These domains are 1.0–1.5 μm in diameter [72], [73]. Each mammalian nucleus contains between one to three of OPT domains that appear during the G1 stage of the cell division, usually close to the nucleoli, and disappear on the S stage [6]. Each OPT domain typically contains 2 or 3 transcription ‘factories’ where bromouridine-triphosphate BrUTP (BrUTP, a specific label, which, being introduced into eukaryotic cells in culture, substitutes for UTP during transcription, thereby providing reliable readout of pre-mRNA for detection by immunochemical methods), is incorporated into nascent transcripts [73]. Two of the transcription factors found in this subnuclear organelle, PTF and Oct1, are known to activate the transcription of genes encoding snRNAs and other ‘processing RNAs’. These proteins bind to proximal and distal sequence elements (PSEs and DSEs) within the promoters and activate transcription by RNA polymerases II or III [73]. Sp1 is the promoter-specific transcription factor that enhances transcription from a variety of genes by binding to GC-rich decanucleotide recognition elements (GC boxes) within the 5′-flanking promoter sequences [74], [75].

**Fig. 7:**
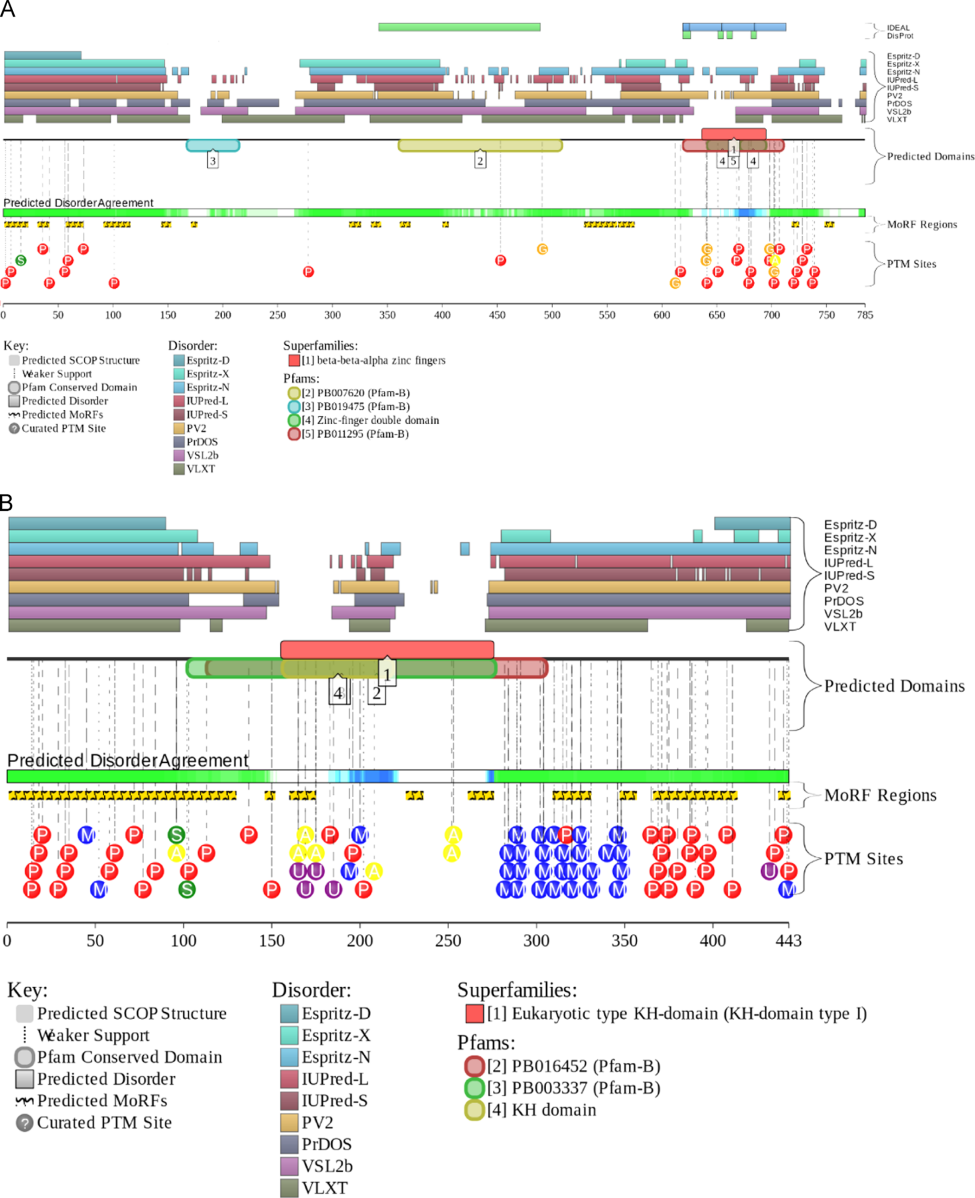

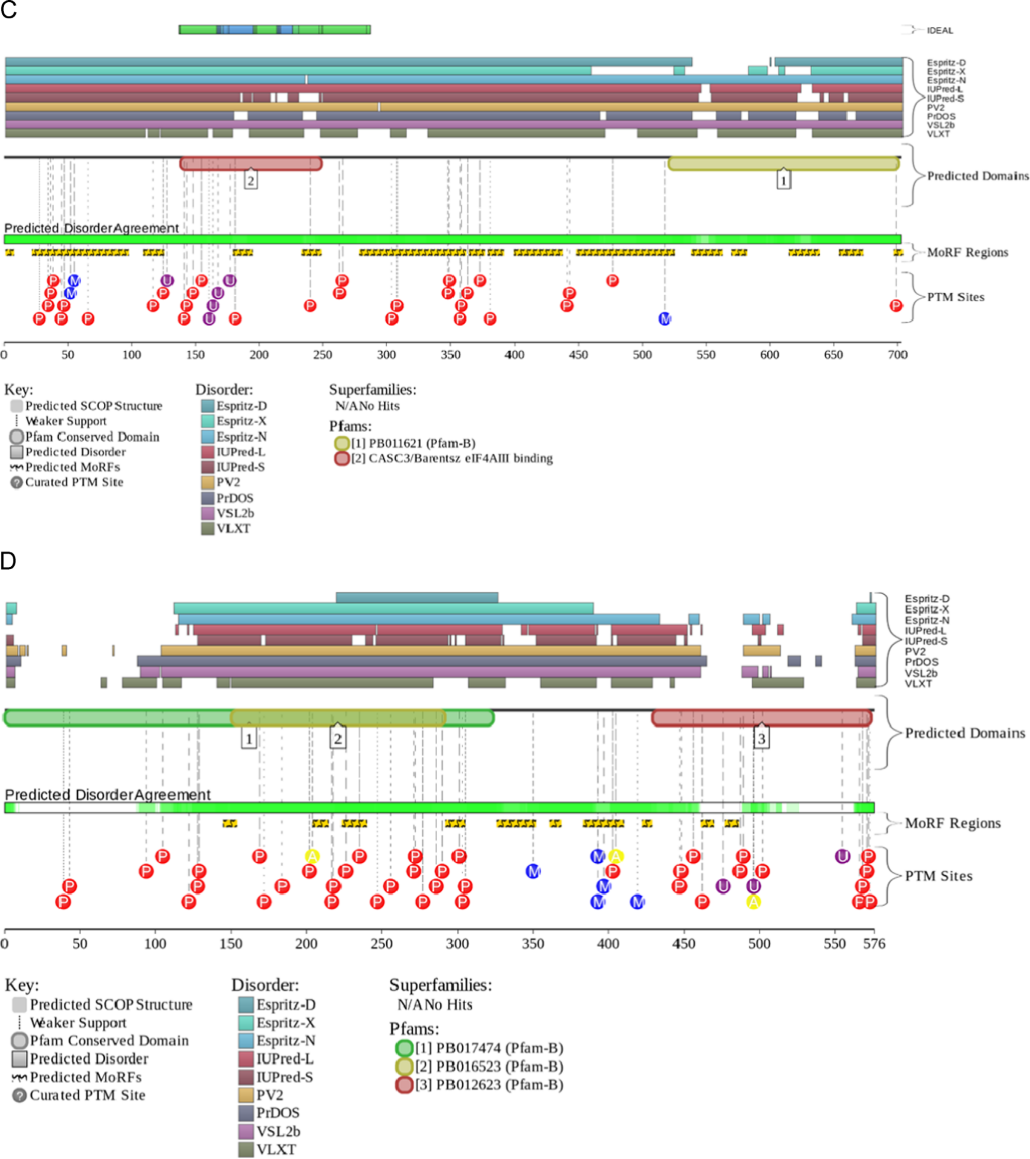

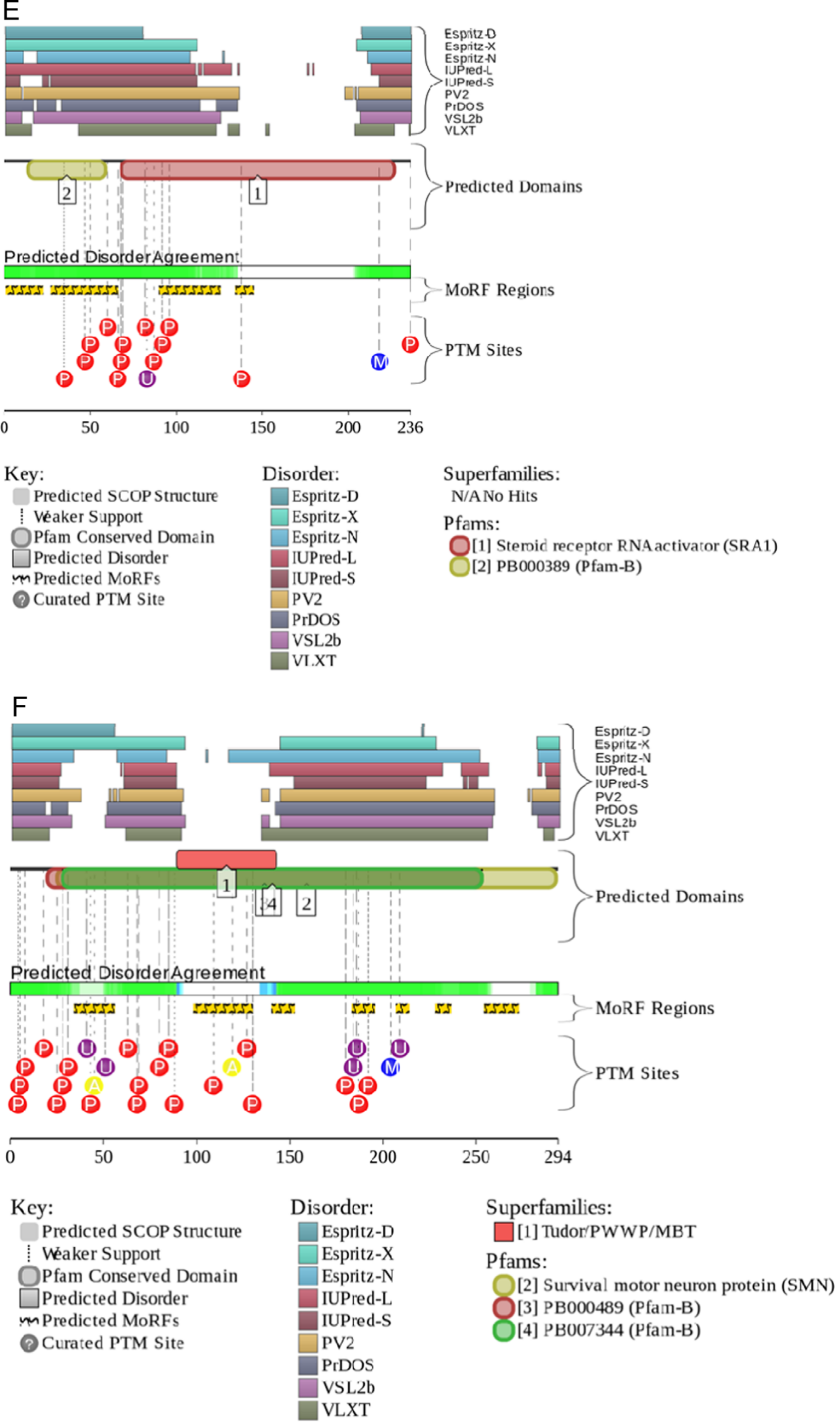
Evaluation of the functional intrinsic disorder propensity of representative human nuclear proteins by D^2^P^2^ database (http://d2p2.pro/) [19]. (A) Sp1 (UniProt ID: P08047). (B) Sam68 (UniProt ID: Q07666). (C) CASC3 (UniProt ID: O15234). (D) Coilin (UniProt ID: P38432). (E) SRA1p (UniProt ID: Q9HD15). (F) SMN (UniProt ID: Q16637).

The level of intrinsic disorder in this nuclear domain is high, since the majority of its constituents are transcription factors; i.e., proteins that are known to be highly disordered in general [76], [77], [78], [79]. In agreement with these general observations, one of the proteins found in the OPT domain, transcription factor Sp1 (UniProt ID: P08047), contains very significant amount of disorder (see Fig. 7A). In fact, the overall disorder content of this protein evaluated by PONDR^®^ VSL2 is 72%. Also, Fig. 7A shows that this transcription factor has multiple disorder-based interaction sites, is full of PTMs and has several functional domains. The DNA-binding domain located at the C-terminus of this transcription factor is known to contain three contiguous Cys_2_-His_2_ zinc finger domains with the consensus sequence Cys-X2-4-Cys-X12-His-X3-His [80]. Structures of these zinc finger domains (residues 619–654, 654–684, and 684–712) in their zinc bound form have been determined by NMR [81]. This analysis revealed that although all of these DNA binding motifs have a canonical fold, they interact with DNA differently [81]. In fact, each of fingers 2 and 3 recognizes four base pairs strictly by using residues at positions −1, 2, 3, and 6 of the recognition helix. However, the interaction mode of finger 1 is quite different and this motif can use only two residues for DNA recognition at positions −1 and 3 of the helix [81]. As a result, in comparison with other Cys_2_-His_2_ zinc fingers, finger 1 has more relaxed sequence and site specificity. Based on these observations it was suggested that this relaxed finger 1 defines the ability of Sp1 to bind various DNA sequences with high affinity [81].

### 4.3 Disorder in SAM68 nuclear bodies (average disorder score 72%)

Similar to the perinucleolar compartment (PNC, see below) SAM68 nuclear bodies are unique structures associated with the surface of nucleoli and involved in the RNA metabolism [6], [82]. They range in size from 0.25 to 1.0 μm in diameter, and there are 1–10 of these bodies per nucleus. The major components of the SAM68 nuclear bodies are the members of a group of RNA-binding proteins that contain a GSG domain (GRP33, Sam68, GLD-1), also known as the signal transduction and activation of RNA (STAR) domain [6]. The founding member of this class of proteins, Src associated in mitosis 68 kDa protein (Sam68, also known as KH domain-containing, RNA-binding, signal transduction-associated protein 1, KHDRBS1), belongs to the heteronuclear ribonucleoprotein particle K (hnRNP K) homology (KH) domain family of RNA-binding proteins [83]. Sam68 is involved in various post-transcriptional regulation events, such as alternative splicing and RNA export [84], [85], [86]. Since Sam68 is involved in protein and RNA binding, this dual activity is reflected in its structure [83]. Protein has the KH domain (residues 171–197) flanked by conserved N- and C-terminal sequences needed for its RNA binding and four proline-rich regions needed for interaction with SH3 and WW domain-containing proteins [83]. These proline-rich protein recognition sequences are flanked by Arg/Gly-rich motifs which can be asymmetrically dimethylated on arginine residues to give the DMA/Gly-rich regions. For human protein, structure is known for the homodimerization domain (residues 97–135) [87] and the tyrosine-rich domain (residues 365–419) in a complex with the armadillo repeat domain of adenomatous polyposis coli (APC) [88]. The unusual topology of the homodimerization domain is indicative of the folding upon binding mechanism, where intrinsically disordered monomers fold at dimerization. In the Sam68-APC complex, only 11 of 55 residues of the Sam 68 tyrosine-rich domain are visible (residues 379–389), indicating that the N- and C-termini of this fragment are disordered. In agreement with these observations, Fig. 7B shows that Sam68 is predicted to be heavily disordered and contain numerous disorder-based interactions regions and numerous sites of posttranslational modifications (PTMs).

### 4.4 Disorder in nuclear speckles (average disorder score 65%)

Nuclear speckles (or interchromatin granule clusters) are the storage sites for the pre-mRNA splicing factors that can be later recruited by RNA polymerase II transcription sites in the nucleoplasm. The cell nucleus contains 25–50 such speckles which are diffusely distributed throughout the nucleoplasm and that include a set of pre-mRNA splicing factors [89]. In *Drosophila*, proteins targeted to the speckled regions were shown to contain an arginine/serine- (RS) rich domain composed of approximately 120 amino acids [89]. One of the proteins associated with the nuclear speckles is cancer susceptibility candidate gene 3 protein (CASC3, 703 residuies, UniProt ID: O15234), which is a typical representative of the IDP world. This protein is predicted to be highly disordered, to contain a large number of PTM sites and several disorder-based molecular recognition regions (see Fig. 7C). Structural information is available for the functional region (residues 137–283) required for the RNA-binding, interaction with MAGOH, localization in nucleus speckles, and to the formation of the splicing-dependent exon junction complex (EJC). Of 146 residues used in crystallization experiment, only central region (residues 172–249) was resolved, whereas both N- and C-termini showed high level of disorder manifested in missing electron density for residues 137–171 and 249–283 [90].

### 4.5 Disorder in chromatin and heterochromatin (average disorder scores 60% and 51%)

The nucleoplasm contains thread-like, coiled, elongated structures, which are chromatin or chromatin fibers. Chromatin is not evenly distributed within the nucleus but is known to be organized into the chromosome territories [6], [91], [92]. During cell division, these fibers take ribbon-like structures and are known as chromosomes (molecules made of DNA and proteins). The chromatin fibers are twisted, fine, anastomose, and uniformly distributed within the nucleoplasm. There are two segments in the chromatin: heterochromatin and euchromatin. Heterochromatin, being present in certain places in the chromatin, is genetically inert and does not take part in transcription. It only replicates late in the S-phase of the cell. Euchromatin, in turn, replicates early, forms the bulk of the chromatin, is genetically active and only a portion takes part in transcription. Chromatin is a way of dynamic storage of long DNA molecules. The primary functions ascribed to chromatin are DNA packaging into a smaller volume to fit in the cell, DNA reinforcement to allow mitosis, preventing DNA damage, and providing means to control DNA replication and gene expression [93]. The dynamics of chromatin structure is tightly regulated through multiple mechanisms such as histone modification, chromatin remodeling, histone variant incorporation, and histone eviction [93].

The major players in the hierarchical packaging of genomic DNA in the eukaryotic nucleus are histone proteins. In fact, the fundamental repeating unit of chromatin is the nucleosome that comprises 146 base pairs of DNA wrapped in 1.7 superhelical turns around a core histone octamer consisting of two dimers of H2A-H2B that serve as molecular caps for the central (H3–H4)_2_ tetramer [94], [95], [96]. At the first level of chromatin organization, nucleosomes are arranged in the specific array, that represents a “beads-on-a-string” fiber with a diameter of 11-nm [95]. The subsequent binding of the linker histone (H1 or H5) transforms the nucleosome arrays into a more condensed 30-nm chromatin fiber, which represents the second structural level of DNA organization [96], [97].

Histones are small, highly basic nuclear proteins that associate with DNA in a specific stoichiometry to form the nucleosome, which further contributes to the formation of the chromatin fiber to package the complete genome within the nucleus. There are five classes of histones in mammals, namely core histones H2A, H2B, H3, H4, and a linker histone H1 (or H5 in avian erythrocytes, which unlike mammalian erythrocytes, have nuclei). Each histone class has various numbers of variants that are expressed in a cellular context-dependent manner. Activity of histones is tightly regulated via the broad range of reversible, enzymatic posttranslational modifications (PTMs), constituting a specific histone code [98], [99], [100], [101], [102]. Since the major function of histones is DNA condensation in chromatin, these proteins are intimately involved in major cellular processes such as DNA damage response, X chromosome inactivation, transcriptional regulation, and even formation of an epigenetic memory [103], [104], [105], [106], [107], [108], [109], [110]. Several diseases and syndromes are related to the dysregulation of histone functions and PTMs [111].

The fact that the histone tails are typical IDPRs is well-established [112]. For example, the N-terminal tails are the most basic regions of the histones that contain no acidic residues, and have very high contents of basic residues [113]. The C-terminal sequences of core histones extend beyond the histone fold and are highly dynamic [95]. The highly dynamic nature of histone tails is visualized by the X-ray structures of nucleosomes, where tail domains appear to sample multiple conformations [95], [114], [115], [116], [117]. The intrinsically disordered nature of the N-terminal “tail” domains (NTDs) of the core histones and the C-terminal tail domains (CTDs) of linker histones, peculiarities of their amino acid compositions, and the role of intrinsic disorder in functioning and posttranslational modifications of these domains were systemized in a review by Hansen et al. [118]

Furthermore, pure histones dissolved in water with no added salt are intrinsically disordered [119], [120], [121], [122], [123], [124], [125], [126], [127]. However, in the presence of salt they adopt folded conformation [121], [122], [123], [124], [125], [126], [127]. This salt-induced refolding is a highly cooperative conformational change that is similar to the transitions observed during the renaturation of unfolded globular proteins [127]. Systematic structural characterization of a sample of histones from calf thymus, representing a mixture of core histones H2A, H2B, H3, and H4, revealed that the bovine core histones are intrinsically disordered proteins [128]. This conclusion was recently extended to >2000 histones from all five histone classes found in ~750 species [129]. Here, the comprehensive computational analysis revealed that the majority of the histone family members were predicted to be mostly disordered, with intrinsic disorder extending far beyond the limits of mentioned NTDs of the core histones and CTDs of linker histones [129].

### 4.6 Disorder in PcG bodies (average disorder score 59%)

PcG bodies are associated with pericentromeric heterochromatin [130]. They got their name from their content, which is polycomb group (PcG) complex containing Ring1, Bmi1, and hPc2 proteins [130]. The number of these domains per cell varies from two to several hundred. PcG bodies are not randomly dispersed, but are clustered into defined areas within the nucleus. They also vary in size (0.2–1.5 μm) and protein composition [6], [130]. It is believed that PcG bodies are required to maintain the transcriptionally repressive state of many genes, including Hox genes, throughout development [131]. PcG proteins can form different complexes (e.g., polycomb repressive complexes 1 and 2, PRC1 and PRC2) which are different in composition and function [132], [133]. For example, PRC1 includes Bmi1, HPH2, PC3, and Ring proteins (Ring1A and Ring1B) [134] and have two major biological functions, binding chromatin to prevent it from being remodeled by ATP-dependent remodeling factors, [135] and serving as an E3 ubiquitin ligase responsible for the mono-ubiquitination of the nucleosomal histone H2A at lysine 119 [134]. On the other hand, PRC2 complex serves as a histone methyltransferase responsible for the methylation of histone H3 at lysine 27 [136], [137].

Bmi1 plays a central role in the assembly of the mammalian PRC1 complex. Although this protein does not have detectable ubiquitin ligase activity, the binding of Bmi1 greatly stimulates the E3 ligase activity of the Ring1B [131], [134]. Human Ring1B (residues 1–336) and a Bmi1 fragment encompassing residues 1–230 were used in the analysis of the structural prerequisites of the Bmi1-Ring1B complex formation [138]. The pre-crystallization treatment of the mixture of these proteins with the protease elastase generated mini Bmi1-Ring1B core that included the N-terminal regions of both proteins (residues 5–101 of Bmi1 and residues 15–114 of Ring1B) [138]. The fact that very large parts of both proteins can be removed by proteolysis indicates that these regions (or their significant fractions) are intrinsically disordered.

### 4.7 Disorder in the nuclear pore (average disorder score 55%)

Since the nucleus is one of the membrane-enclosed cellular organelles, it has an outer structure (see Fig. 1), the nuclear envelope, which separates the contents of the nucleus from the cytoplasm and known to disappear during cell division. The nuclear envelope is composed of two layers: an outer membrane (which is contiguous with the endoplasmic reticulum) and an inner membrane (also known as nuclear lamina), each of about 75–90 Å thick and lipoproteinaceous in nature. The space between these two membranes is known as the perinuclear space [139]. Both the nuclear envelope and the nuclear lamina enable the exchange of ions between the nucleus and cytoplasm, and in some cell types (such as salivary glands) these two structures work as a barrier for the diffusion of substances and some ions, such as potassium(K^+^), sodium (Na^+^) and chlorine (Cl^–^) [140], [141], [142].

The integrity of the nuclear envelope is interrupted by the presence of nuclear pores, large, multichain proteinaceous machines, known as the nuclear pore complexes. The number of pores found in the nuclear envelope depends on the species and the type of the cell. The pores are arranged hexagonally along the membrane. It was estimated that these pores cover 10% of the surface of the nucleus in mammalian cells. In some cases, this pore complex remains covered by a thin membrane that may serve for selective permeability, which depends on both the cell type and its metabolic state [143], [144], [145]. Nuclear pore is a large proteinaceous machine (124 MDa in mammals) that crosses the nuclear envelope and contains approximately 30 different protein components (nucleoporins), each in multiple copies [145]. About half of the nucleoporins are ordered transmembrane proteins that typically contain either an α-solenoid or a β-propeller fold, or, for multidomain nucleoporins, both folds in separate structural domains. The other half nucleoporins belongs to the category of IDPs; i.e., many these nucleoporins are characterized by extended structure, high conformational flexibility, and lack of ordered secondary structure [146], [147], [148], [149]. Careful structural analysis of gate-forming nucleoporins containing large IDPRs with multiple phenylalanine–glycine repeats (FG domains) revealed that these IDPs could be grouped into at least two distinct categories of intrinsically disordered structures. Some nucleoporins adopt more collapsed configurations and are characterized by low charge contents. Others are highly charged and adopt more dynamic, extended coil conformations. Interestingly, several FG nucleoporins feature both types of structures in a bimodal distribution along their polypeptide chain [148].

### 4.8 Disorder in Cajal body (average disorder score 54%)

Cajal bodies (CBs) are roughly spherical 0.1–2.0 µM structures numbering one to five per nucleus. They are not easily seen in all cell types, but are prominent in highly active cells such as cancer cells or neurons [150], [151]. CBs also known as nucleolar accessory bodies or coiled bodies are conserved from plant to mammals. The name “coiled bodies” was given to these suborganelles because of the presence of the coiled threads of the marker protein, coilin [150], [151]. Being able to concentrate the components of several nuclear processes, CBs are thought to be responsible for the increased efficiency of the corresponding processes (e.g., assembly of U snRNPs, some of which eventually form the RNA splicing machinery, or spliceosome) [151]. Fig. 7D shows that coilin is predicted to be disordered and enriched in PTM sites and disorder-based recognition motifs. In agreement with these predictions, structural analysis of the coilin from *Arabidopsis thaliana* revealed that this protein has a loosely ordered N-terminal domain (residues 1–117), a highly disordered central domain (residues 117–350) and a loosely ordered C-terminal region (residues 370–608) containing the Tudor-like domain [152].

### 4.9 Disorder in perinucleolar compartment (average disorder score 52%)

The perinucleolar compartment (PNC) is irregularly shaped nuclear body, ranging from 0.25 to 4.0 µm in length, and is associated with the periphery of the nucleolus [153], [154], [155]. It contains several short RNAs which are RNA polymerase III transcripts (such as Y RNAs, MRP RNA, and RNase P H1 RNA) and the polypyrimidine tract-binding protein, heterogeneous nuclear ribonucleoprotein I (hnRNP I) [153]. Despite intensive efforts, the structural information on the full-length hnRNP I (UniProt ID: P31943) is still missing. Although several fragments of this protein (its RNA recognition motifs, RRMs) were structurally characterized by NMR (e.g., RRM1, residues 55–147 [156], RRM2, residues 147–301 [156], RRM3-RRM4, residues 324–531 [157]) or X-ray crystallography (RRM2, residues 156–286 [158]), all structures contain long disordered regions. This is in agreement with computational characterization of intrinsic disorder in human hnRNP I, which is predicted to have long disordered N-tail and long disordered linkers connecting RRMs. This “beads-on-a-string” structural organization defines the capability of this proteins for multivalent binding and extends the search area for this multivalent interaction.

### 4.10 Disorder in nucleolus (average disorder score 44%)

Besides the chromatin, an important organelle which is clearly visible in the nucleoplasm is the nucleolus. The nucleolus is a dense, spherical, acidophilic structure. Its size is related to the ribosomal demands of the cell. Cells with no synthetic activities (such as spermatozoids or muscle cells) have smaller or no nucleolus, whereas cells producing large amounts of proteins (and, thereby, requiring more ribosomes) have large size nucleolus [159]. The main function of the nucleolus is the biogenesis of ribosomal subunits, which are later taken to the cytoplasm for the translation of RNA. The typical nucleolus is composed of 3 regions: fibrillar center, which contains the genes of the nuclear organizer of chromosomes, the fibrillar components which are responsible for RNA synthesis, whereas the cortical granular components – the outermost regions – are in charge of the maturation of pre-ribosomal particles [160].

The nucleolar proteins represent the largest subset of human nuclear proteins analyzed in this study. One of the best studied IDPs found in this compartment is an inhibitor of cyclin dependent protein kinases (Cdks) p27^kip1^ [49], [161], [162], [163], [164], [165], [166], [167]. Being essentially unfolded in its unbound state, p27^kip1^ gains structure at interaction with cyclin A/Cdk2 complex [41], [120], [121], [122], [123], [124], [125], [126]. The flexibility and lack of structure are key features of the p27^kip1^-based signaling conduit [162], [163], [164]. The p27^kip1^ chain wraps around the cyclin A/Cdk2 complex and interacts with features at multiple locations, including some on opposite sides of the complex [168]. Such interaction mode supports a fully disordered state in uncomplexed p27^Kip1^. This type of coupled folding and binding was described for some other IDPs [169], [170], [171]. Lack of structure in the unbound state facilitates the unzippering of complexes, [7] thereby allowing part of a complex to separate while maintaining other interactions. The flexibility of this tethered disordered segment further enables p27^kip1^ to fold back and thereby accelerates phosphorylation via a unimolecular mechanism [164].

### 4.11 Disorder in PML body (average disorder score 37%)

PML bodies, also referred to as ND10, PODs (PML oncogenic domains) and Kr bodies, vary in size from 0.3 µm to 1.0 µm in diameter [172]. The average mammalian nucleus can contain between 10 and 30 of these structures. Although PML bodies have been found juxtaposed to other nuclear structures such as nuclear gems and Cajal bodies, the significance of this association is unknown. Neither chromatin nor RNA is found within the central core of these bodies, but newly synthesized RNA is associated with their periphery [173]. The human MHC locus on chromosome 6 has been shown to associate preferentially with PML bodies [174]. The PML body contains satellite DNA and several proteins such as Sp100, SUM01, HAUSP, HP1, BRCA1, ATRX, DAXX, and, the promyelocytic leukemia (PML) protein [29], [175]. This subnuclear organelles are believed to be involved in the re-establishment of the condensed heterochromatic state on late-replicated satellite DNA [175].

One of the well-characterized IDPs found in PML bodies is an important tumor suppressor protein BRCA-1, which is known to participate in many cellular pathways, such as transcription, apoptosis, and DNA repair, through direct or indirect interaction with a variety of partners [176]. A canonical isoform of this protein is a 1863 residues-long polypeptide that contain a long intrinsically disordered central region [177], flanked by ordered domains at the two termini, an N-terminal RING finger domain of 103 residues, and C-terminus located two tandem copies of the BRCA1 C-terminal domain with a total of 218 residues making up the two domains. The long central IDPR of BRCA1 contains numerous molecular recognition domains for DNA and several protein-binding partners [178], [179]. It is known that intrinsic disorder plays a unique role in alternatively splicing-based modulation of the BRCA1 functionality. In fact, alternatively splicing (AS) predominantly affect the long central IDPR of BRCA1, and different AS isoforms described for BRCA1 lack various functional regions [178], [179]. The removal of specific functional regions in various AS isoforms creates diverse functional profiles for the transcribed gene products. This AS-based functional re-profiling of IDPs was attributed to the facts that regulatory and signaling elements in IDPRs usually contain just a few residues, and that the density of these functionally important segments within the IDPRs is high [178], [179].

### 4.12 Disorder in RNA polymerase II transcription machinery (average disorder score 27%)

RNA polymerase II (Pol II) is a proteinaceous machine that catalyzes the transcription of DNA to synthesize precursors of mRNA and most snRNAs and microRNAs [180], [181]. In humans, the core enzyme has 12 subunits, RBP1-RBP12, which range in size from ~6 to 200 kDa [182]. The level of evolutionary conservation of this machine is so high, that many yeast Pol II subunits can be replaced with their mammalian counterparts without deleterious effects on cell function [182], [183], [184], [185], [186]. The core Pol II is incapable of promoter recognition and requires a wide range of general transcription factors and regulatory proteins to bind to upstream gene promoters and begin transcription. Therefore, the function competent form of this enzyme, which is known as RNA polymerase II holoenzyme, consists of core Pol II, a subset of general transcription factors, and regulatory proteins known as SRB proteins [182].

A peculiar protein found in association with the RNA polymerase II transcription machinery is the steroid receptor activator RNA protein (SRA1p). SRA1p is the translation product of the bi-functional steroid receptor activator RNA 1 (SRA1), which is a large RNA transcript that can be processed as a messenger RNA, but also acts as a long non-coding RNA (lncRNA), possibly serving as a component of the steroid receptor coactivator-1 complex [187], [188]. SRA1p can be divided into four specific regions: (i) a basic, intrinsically disordered N-terminal tail (residues 1–64, 7 kDa, p*I*~9.5); (ii) an intrinsically disordered, proline-rich (40%) linker region (residues 65–106); (iii) an RNA-binding, α-helical, 103 residue-long acidic domain (residues 107–209, 14.7 kDa, p*I*=6), which is folded similar to the PRP18 splicing factor; and (iv) a long disordered C-tail (residues 210–236) [188]. In agreement with this structural description derived from the NMR analysis of this protein, Fig. 7E shows that SRA1p is predicted to have long disordered tails, disorder-based binding regions and multiple PTM sites.

### 4.13 Disorder in gems (average disorder score 25%)

Gems contain the survival motor neuron (SMN) protein encoded by the *SMN1* gene, which is frequently mutated or deleted in spinal muscular atrophy [189], [190]. SMN forms a complex with Gemins 2–7, interacts with Sm, Sm-like proteins, RNA helicase A, and hnRNP R, Q, and U [191], plays a critical role in snRNP biogenesis [192], [193], and is implicated in the assembly of short nucleolar ribonucleprotein (snoRNP) particles [194] and the RNA polymerase II transcription and processing machinery [190], [195]. SMN is predicted to be a highly disordered protein (see Fig. 7F). In agreement with these predictions, the structural information for the whole protein is not available as of yet. However, a short fragment corresponding to the N-terminal α-MoRF (residues 26–62) was co-crystallized with Gemin 2 [196]. Also, structural information is available for the Tudor domain of this protein (residues 84–147) [197], which is the only relatively long region of predicted order in this protein (see Fig. 7F). As typical for many regulatory IDPs [198], [199], the SMN protein is heavily phosphorylated.

### 4.14 Intrinsically disordered proteins as critical constituents of nuclear domains and major controllers of assembly and disassembly of these organelles

Recently, it has been proposed that IDPs may play an important role in driving the intracellular liquid–liquid phase separations generating various membrane-less organelles [200]. It has been emphasized that these membrane-less organelles are formed via the colocalization of molecules at high concentrations within a small cellular micro-domain. Among considered examples of such organelles were PML bodies or nuclear dots, or PODs, perinucleolar compartment (PNC), the Sam68 nuclear body (SNB), paraspekles, nuclear speckles or interchromatin granule clusters, nucleoli, processing bodies, germline P granules, Cajal bodies (CBs), centrosomes, and stress granules [200], many of which are subnuclear organelles considered in our study. Being devoid of membranes, these organelles are highly dynamic, with their components being in direct contact with the surrounding nucleoplasm or cytoplasm [201], [202]. Such membrane-less organelles are only slightly denser than the bulk intracellular/intranuclear fluid, are characterized by high level of internal dynamics, and therefore they can be considered as liquid-droplet phases of the nucleoplasm/cytoplasm [203], [204], [205], [206], [207], [208]. Being found in different cellular and nuclear locations and being composed of rather different proteins and nucleic acids, these organelles are formed via a common mechanism related to the intracellular phase transitions [209]. These phase transitions are driven by the effects of macromolecules on the structure and solvent properties of water and are related to the high concentrations of macromolecular solutes, since at low macromolecule concentrations, the solution exists as a single phase, whereas at high concentrations, phase separation occurs [210]. Earlier study revealed that many proteins responsible for the formation of the cytoplasmic or nucleoplasmic membrane-less organelles are in fact intrinsically disordered [200]. It was also hypothesized that because of the IDPs are known to be engaged in various weak interactions of different physico-chemical nature and because of these proteins are commonly seen in different cytoplasmic and nuclear membrane-less organelles, IDPs might serve as perfect regulators and controllers of the formation of these organelles via the aforementioned phase separation [200]. Our current work provides additional support to this idea and shows that many subnuclear organelles in human cells are enriched in IDPs and hybrid proteins possessing ordered and disordered domains and that human nuclear proteins are very promiscuous binders possessing both large quantities of potential disorder-based interaction sites and the ability of a single such site to be involved in large number of interactions.
