# Supplementary material for: Intrinsically disordered proteins in the nucleus of human cells
Source: Biochem Biophys Rep. 2015 Mar 24;1:33–51. doi: 10.1016/j.bbrep.2015.03.003 (PMC5668563; doi:10.1016/j.bbrep.2015.03.003)
Supplement: Supplementary file 1 — Supplementary data [file mmc1.doc]

**Supplementary materials**

**Intrinsically disordered proteins in the nucleus of human cells**

**Telma Frege,^1,2^ and Vladimir N. Uversky^1,3-4,*^**

*^1^Department of Molecular Medicine, Morsani College of Medicine, University of South Florida, Tampa, FL 33612, USA*

*^2^GenomeNext LLC, 175 South 3rd Street, Suite 200, Columbus OH 43215, USA*

*^3^USF Health Byrd Alzheimer's Research Institute, Morsani College of Medicine, University of South Florida, Tampa, Florida 33612, USA*

*^4^Department of Biology, Faculty of Science, King Abdulaziz University, P.O. Box 80203, Jeddah 21589, Kingdom of Saudi Arabia*

*^5^Institute for Biological Instrumentation, Russian Academy of Sciences, 142290 Pushchino, Moscow Region, Russia*

*^6^Laboratory of Structural Dynamics, Stability and Folding of Proteins, Institute of Cytology, Russian Academy of Sciences, St. Petersburg, Russian Federation*

Figure S1. A pipeline designed to study human sub-nuclear proteins.

**
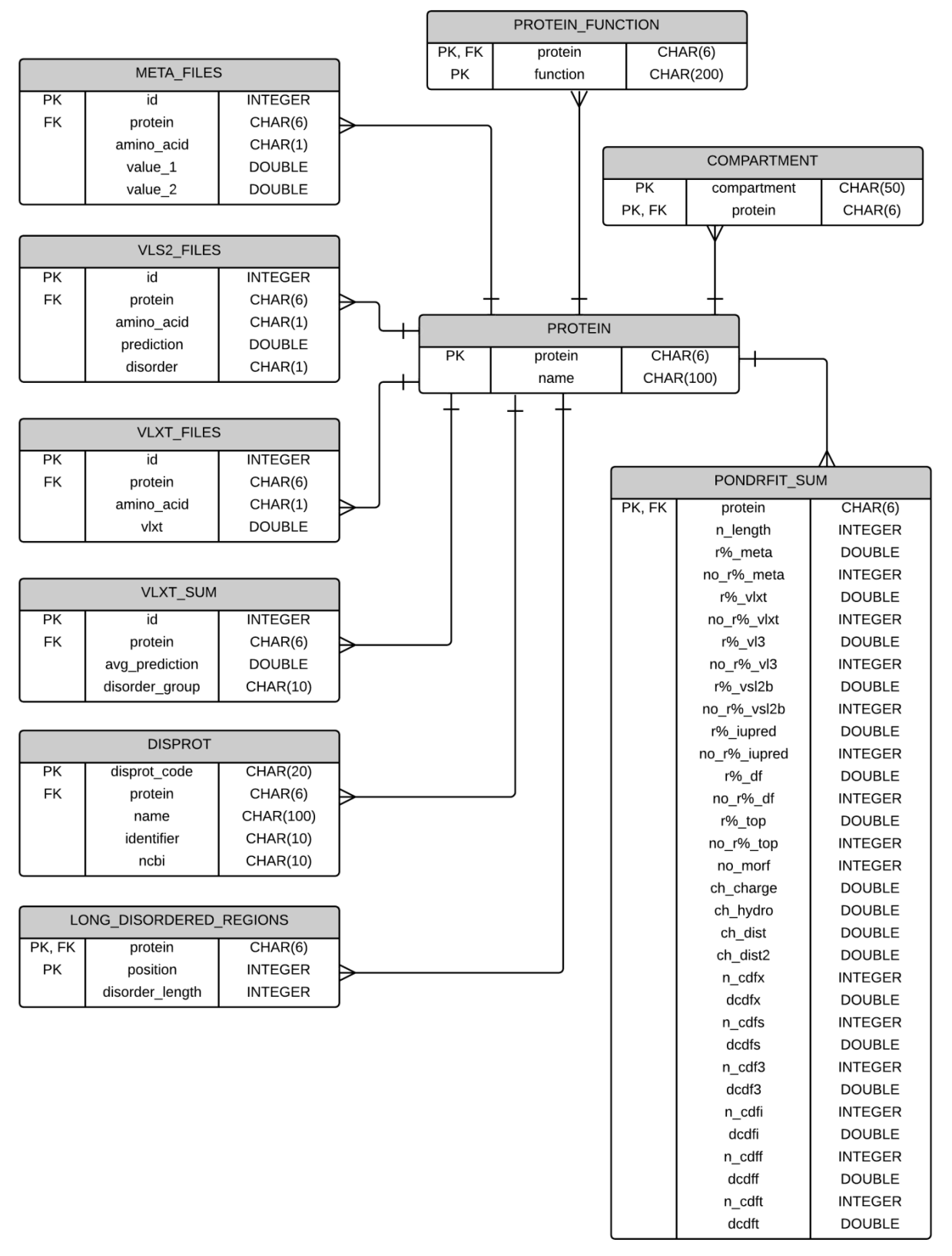
**

**Figure S2**. Entity-relationship diagram used in this study.


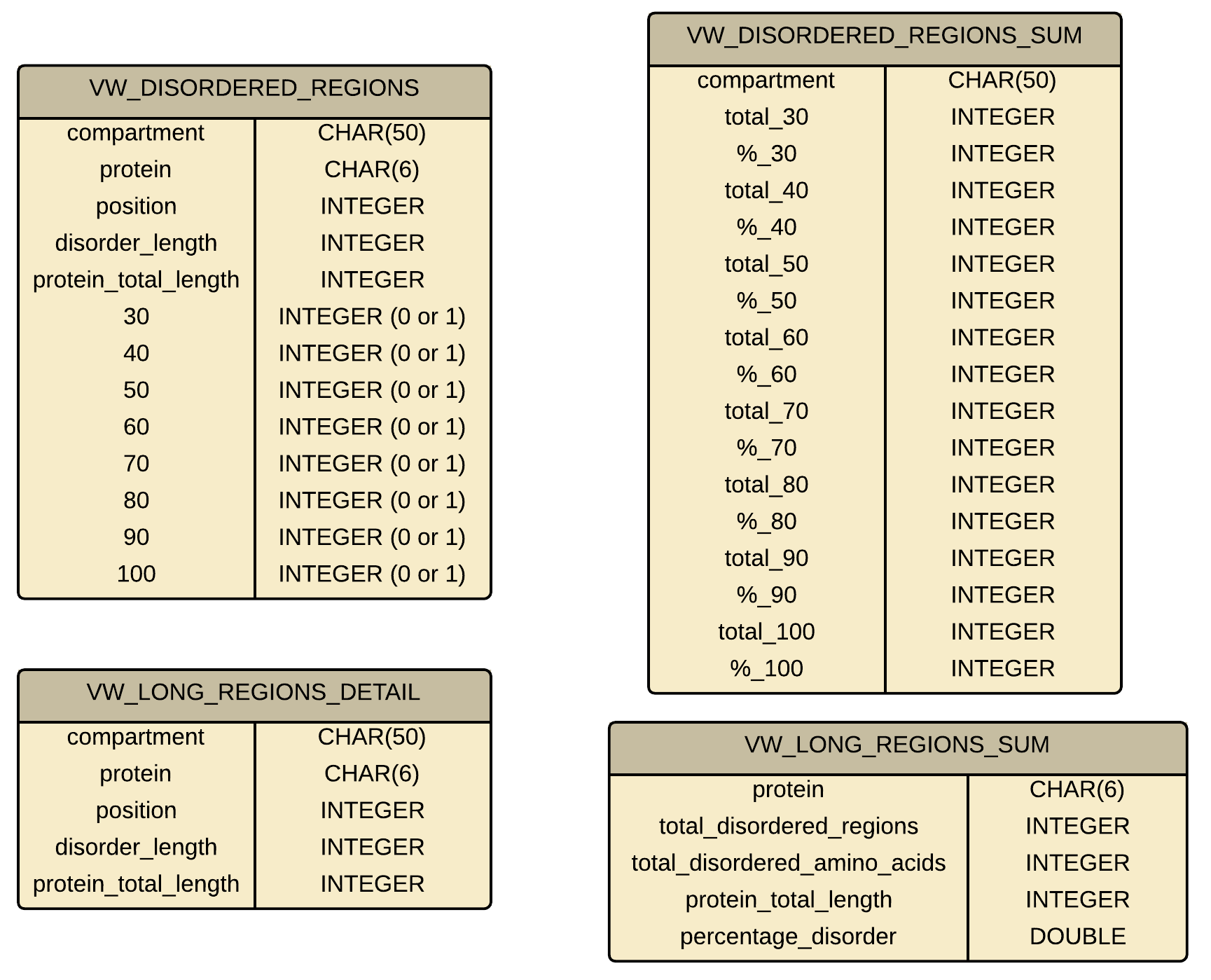


Figure S3. Views created from the tables.


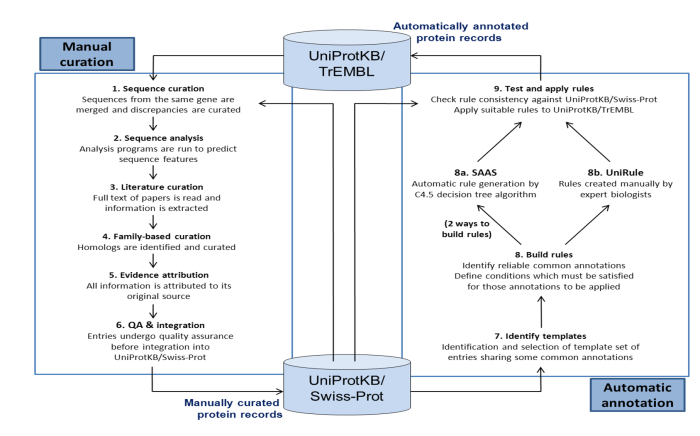


Figure S4. UniProt approach to curate proteins.[55]

## Table S1. Details of disorder prediction for all 185 human nuclear proteins analyzed in this study

| **Compartment** | **Protein** | **Total # disordered regions** | **Total # disordered residues** | **Protein length** | **% disorder** | **Total # AIBSs** | **Length of longest and shortest AIBSs** |
| --- | --- | --- | --- | --- | --- | --- | --- |
| Cajal Body | Q8NC51 | 1 | 387 | 408 | 95% | 13 | 34, 9 |
| Cajal Body | Q14978 | 1 | 640 | 699 | 92% | 17 | 39, 6 |
| Cajal Body | P33240 | 1 | 375 | 577 | 65% | 8 | 136, 6 |
| Cajal Body | Q16637 | 1 | 114 | 294 | 39% | 7 | 32, 7 |
| Cajal Body | Q8TEQ6 | 2 | 312 | 1508 | 21% | 9 | 26, 6 |
| Cajal Body | P09874 | 1 | 110 | 1014 | 11% | 5 | 12, 6 |
|  | | | | | | | |
| Chromatin | Q9HD42 | 1 | 118 | 196 | 60% | 3 | 18, 6 |
|  | | | | | | | |
| Cleavage body | P06454 | 1 | 111 | 111 | 100% | 3 | 42, 14 |
| Cleavage body | P33240 | 1 | 375 | 577 | 65% | 8 | 136, 6 |
|  | | | | | | | |
| Gem | Q16637 | 1 | 114 | 294 | 39% | 7 | 32, 7 |
| Gem | Q8TEQ6 | 2 | 312 | 1508 | 21% | 9 | 26, 6 |
| Gem | Q9UHI6 | 1 | 125 | 824 | 15% | 9 | 36, 6 |
|  | | | | | | | |
| Heterochomatin | P52926 | 1 | 109 | 109 | 100% | 3 | 33, 16 |
| Heterochomatin | P12270 | 8 | 4579 | 4726 | 97% | 30 | 139, 6 |
| Heterochomatin | P17676 | 1 | 331 | 345 | 96% | 9 | 23, 7 |
| Heterochomatin | P51608 | 2 | 454 | 486 | 93% | 12 | 62, 9 |
| Heterochomatin | P46013 | 6 | 3004 | 3256 | 92% | 97 | 57, 6 |
| Heterochomatin | Q96DN6 | 1 | 920 | 1003 | 92% | 23 | 75, 9 |
| Heterochomatin | O00257 | 2 | 944 | 1120 | 84% | 13 | 46, 8 |
| Heterochomatin | Q8TEK3 | 3 | 1335 | 1739 | 77% | 36 | 95, 7 |
| Heterochomatin | O15525 | 1 | 115 | 162 | 71% | 4 | 18, 6 |
| Heterochomatin | O60675 | 1 | 111 | 156 | 71% | 3 | 18, 6 |
| Heterochomatin | P49716 | 1 | 181 | 269 | 67% | 7 | 53, 7 |
| Heterochomatin | Q5VTD9 | 2 | 217 | 330 | 66% | 2 | 18, 13 |
| Heterochomatin | P25440 | 2 | 519 | 801 | 65% | 17 | 44, 6 |
| Heterochomatin | P38398 | 8 | 2420 | 3726 | 65% | 44 | 32, 6 |
| Heterochomatin | Q9NS56 | 3 | 672 | 1045 | 64% | 22 | 37, 7 |
| Heterochomatin | Q8TBE0 | 2 | 489 | 780 | 63% | 13 | 77, 6 |
| Heterochomatin | Q9P267 | 3 | 874 | 1494 | 59% | 34 | 128, 6 |
| Heterochomatin | P46100 | 3 | 1418 | 2492 | 57% | 40 | 36, 6 |
| Heterochomatin | Q00613 | 1 | 272 | 529 | 51% | 6 | 75, 16 |
| Heterochomatin | Q9UER7 | 1 | 380 | 740 | 51% | 18 | 39, 10 |
| Heterochomatin | O95243 | 1 | 290 | 580 | 50% | 4 | 23, 6 |
| Heterochomatin | Q9UIS9 | 1 | 274 | 605 | 45% | 8 | 31, 7 |
| Heterochomatin | Q6KC79 | 1 | 1113 | 2804 | 40% | 34 | 58, 6 |
| Heterochomatin | Q9UKS7 | 2 | 209 | 526 | 40% | 14 | 19, 6 |
| Heterochomatin | O15164 | 1 | 405 | 1050 | 39% | 9 | 122, 10 |
| Heterochomatin | Q13415 | 1 | 337 | 861 | 39% | 8 | 43, 7 |
| Heterochomatin | Q96EB6 | 2 | 289 | 747 | 39% | 14 | 73, 6 |
| Heterochomatin | Q9UBC3 | 2 | 330 | 853 | 39% | 8 | 43, 8 |
| Heterochomatin | Q9UIG0 | 3 | 544 | 1483 | 37% | 22 | 58, 6 |
| Heterochomatin | Q13416 | 1 | 216 | 577 | 37% | 6 | 21, 6 |
| Heterochomatin | Q9NRL2 | 3 | 567 | 1556 | 36% | 14 | 33, 6 |
| Heterochomatin | P26358 | 2 | 535 | 1616 | 33% | 19 | 30, 6 |
| Heterochomatin | Q7Z3K3 | 3 | 449 | 1410 | 32% | 19 | 31, 6 |
| Heterochomatin | Q9NPF5 | 1 | 120 | 467 | 26% | 11 | 26, 8 |
| Heterochomatin | Q9UK53 | 2 | 220 | 844 | 26% | 7 | 13, 8 |
| Heterochomatin | Q92769 | 1 | 120 | 488 | 25% | 2 | 25, 18 |
| Heterochomatin | Q14739 | 1 | 147 | 615 | 24% | 4 | 25, 10 |
| Heterochomatin | Q9NRZ9 | 1 | 195 | 838 | 23% | 3 | 19, 14 |
| Heterochomatin | Q9Y2K7 | 4 | 528 | 2324 | 23% | 6 | 34, 6 |
| Heterochomatin | Q13422 | 1 | 107 | 519 | 21% | 7 | 60, 11 |
| Heterochomatin | P17844 | 2 | 278 | 1842 | 15% | 5 | 13, 6 |
| Heterochomatin | Q13263 | 2 | 226 | 1670 | 14% | 12 | 54, 6 |
| Heterochomatin | Q96FC9 | 1 | 163 | 970 | 17% | 7 | 14, 6 |
|  | | | | | | | |
| Nuclear pore complex | P12270 | 8 | 4579 | 4726 | 97% | 30 | 139, 6 |
| Nuclear pore complex | P49790 | 2 | 1277 | 1475 | 87% | 36 | 29, 6 |
| Nuclear pore complex | Q9Y6D9 | 2 | 591 | 718 | 82% | 11 | 14, 6 |
| Nuclear pore complex | P35658 | 2 | 1563 | 2090 | 75% | 33 | 53, 6 |
| Nuclear pore complex | Q9UKX7 | 2 | 331 | 468 | 71% | 10 | 38, 6 |
| Nuclear pore complex | Q9BZW7 | 2 | 491 | 698 | 70% | 11 | 12, 6 |
| Nuclear pore complex | P37198 | 1 | 328 | 522 | 63% | 11 | 16, 6 |
| Nuclear pore complex | P52948 | 4 | 958 | 1817 | 53% | 24 | 30, 6 |
| Nuclear pore complex | Q8NFH5 | 1 | 170 | 326 | 52% | 6 | 19, 7 |
| Nuclear pore complex | Q9HC62 | 2 | 298 | 589 | 51% | 5 | 19, 9 |
| Nuclear pore complex | P49792 | 8 | 1573 | 3224 | 49% | 33 | 33, 6 |
| Nuclear pore complex | P46060 | 1 | 157 | 587 | 27% | 2 | 50, 29 |
| Nuclear pore complex | Q53GS7 | 1 | 136 | 698 | 19% | 11 | 25, 6 |
| Nuclear pore complex | Q99567 | 1 | 128 | 741 | 17% | 2 | 11, 10 |
| Nuclear pore complex | P24386 | 1 | 104 | 653 | 16% | 7 | 23, 7 |
|  | | | | | | | |
| Nuclear Speckles (splicing) | O15234 | 1 | 703 | 703 | 100% | 15 | 84, 7 |
| Nuclear Speckles (splicing) | Q05195 | 1 | 206 | 221 | 93% | 7 | 18, 8 |
| Nuclear Speckles (splicing) | P18583 | 3 | 2117 | 2426 | 87% | 43 | 142, 6 |
| Nuclear Speckles (splicing) | Q15532 | 1 | 358 | 418 | 86% | 8 | 121, 6 |
| Nuclear Speckles (splicing) | O60885 | 2 | 1108 | 1362 | 81% | 31 | 105, 7 |
| Nuclear Speckles (splicing) | Q9Y618 | 7 | 2032 | 2525 | 80% | 55 | 136, 7 |
| Nuclear Speckles (splicing) | O75400 | 3 | 577 | 957 | 60% | 16 | 27, 8 |
| Nuclear Speckles (splicing) | Q9P0U4 | 1 | 328 | 656 | 50% | 11 | 31, 6 |
| Nuclear Speckles (splicing) | P51531 | 3 | 713 | 1590 | 45% | 23 | 66, 6 |
| Nuclear Speckles (splicing) | Q9BY77 | 1 | 165 | 421 | 39% | 6 | 32, 9 |
| Nuclear Speckles (splicing) | Q13148 | 1 | 157 | 414 | 38% | 5 | 32, 6 |
| Nuclear Speckles (splicing) | Q6PJP8 | 2 | 249 | 1040 | 24% | 16 | 20, 6 |
|  | | | | | | | |
| Nucleolus | P46527 | 1 | 198 | 198 | 100% | 8 | 26, 8 |
| Nucleolus | Q9NZM5 | 2 | 470 | 478 | 98% | 10 | 34, 6 |
| Nucleolus | Q8NC51 | 1 | 387 | 408 | 95% | 13 | 34, 9 |
| Nucleolus | Q01844 | 2 | 593 | 656 | 90% | 14 | 75, 8 |
| Nucleolus | O94842 | 3 | 542 | 621 | 87% | 17 | 46, 6 |
| Nucleolus | P38159 | 1 | 334 | 391 | 85% | 9 | 72, 8 |
| Nucleolus | P17480 | 3 | 643 | 764 | 84% | 15 | 64, 6 |
| Nucleolus | Q9UHA3 | 1 | 133 | 163 | 82% | 1 | 9 |
| Nucleolus | P23246 | 2 | 553 | 707 | 78% | 13 | 72, 10 |
| Nucleolus | Q9UMY1 | 2 | 403 | 514 | 78% | 8 | 49, 8 |
| Nucleolus | O15265 | 2 | 685 | 892 | 77% | 23 | 60, 8 |
| Nucleolus | Q03188 | 1 | 708 | 943 | 75% | 24 | 39, 8 |
| Nucleolus | Q16629 | 1 | 171 | 238 | 72% | 5 | 53, 8 |
| Nucleolus | P07910 | 1 | 218 | 306 | 71% | 5 | 31, 6 |
| Nucleolus | Q92541 | 2 | 491 | 710 | 69% | 15 | 23, 6 |
| Nucleolus | Q9BXS6 | 1 | 300 | 441 | 68% | 13 | 73, 6 |
| Nucleolus | Q9Y580 | 1 | 182 | 266 | 68% | 4 | 35, 12 |
| Nucleolus | Q99848 | 2 | 360 | 612 | 59% | 10 | 25, 7 |
| Nucleolus | P50914 | 1 | 124 | 215 | 58% | 2 | 65, 30 |
| Nucleolus | Q92878 | 4 | 1534 | 2624 | 58% | 12 | 14, 6 |
| Nucleolus | P09038 | 1 | 163 | 288 | 57% | 5 | 71, 9 |
| Nucleolus | Q99459 | 3 | 459 | 802 | 57% | 17 | 63, 6 |
| Nucleolus | O96004 | 1 | 116 | 215 | 54% | 7 | 31, 8 |
| Nucleolus | P26373 | 1 | 113 | 211 | 54% | 5 | 27, 12 |
| Nucleolus | P09651 | 1 | 196 | 372 | 53% | 8 | 10, 6 |
| Nucleolus | Q8N554 | 2 | 317 | 614 | 52% | 10 | 73, 7 |
| Nucleolus | P49711 | 4 | 745 | 1454 | 51% | 14 | 31, 6 |
| Nucleolus | P52272 | 1 | 369 | 730 | 51% | 7 | 19, 6 |
| Nucleolus | Q9UER7 | 1 | 380 | 740 | 51% | 18 | 39, 10 |
| Nucleolus | P51532 | 3 | 817 | 1647 | 50% | 27 | 211, 6 |
| Nucleolus | Q03468 | 3 | 706 | 1439 | 49% | 24 | 36, 6 |
| Nucleolus | P22626 | 1 | 171 | 353 | 48% | 5 | 10, 6 |
| Nucleolus | Q02878 | 1 | 136 | 288 | 47% | 3 | 37, 6 |
| Nucleolus | P54132 | 5 | 1274 | 2834 | 45% | 16 | 26, 6 |
| Nucleolus | Q13151 | 1 | 138 | 305 | 45% | 3 | 11, 9 |
| Nucleolus | P51116 | 1 | 296 | 673 | 44% | 9 | 47, 6 |
| Nucleolus | Q9NW13 | 2 | 335 | 759 | 44% | 15 | 55, 6 |
| Nucleolus | P11487 | 1 | 103 | 239 | 43% | 3 | 28, 6 |
| Nucleolus | Q86US8 | 1 | 612 | 1419 | 43% | 19 | 29, 6 |
| Nucleolus | O95347 | 2 | 499 | 1197 | 42% | 9 | 25, 6 |
| Nucleolus | Q14692 | 3 | 544 | 1282 | 42% | 21 | 45, 7 |
| Nucleolus | Q13206 | 1 | 362 | 875 | 41% | 10 | 26, 6 |
| Nucleolus | Q9GZR7 | 3 | 351 | 859 | 41% | 12 | 57, 6 |
| Nucleolus | Q13601 | 1 | 154 | 381 | 40% | 6 | 14, 6 |
| Nucleolus | P51114 | 1 | 251 | 621 | 40% | 9 | 43, 6 |
| Nucleolus | Q96GQ7 | 2 | 317 | 796 | 40% | 8 | 45, 8 |
| Nucleolus | Q16665 | 1 | 319 | 826 | 39% | 12 | 30, 9 |
| Nucleolus | Q99729 | 1 | 130 | 332 | 39% | 8 | 31, 8 |
| Nucleolus | Q9BXY0 | 1 | 118 | 300 | 39% | 3 | 30, 18 |
| Nucleolus | Q9H8H2 | 2 | 315 | 851 | 37% | 10 | 89, 7 |
| Nucleolus | Q9H2G4 | 1 | 244 | 693 | 35% | 9 | 59, 9 |
| Nucleolus | P15924 | 2 | 932 | 2871 | 32% | 16 | 29, 6 |
| Nucleolus | P36578 | 1 | 138 | 427 | 32% | 5 | 52, 9 |
| Nucleolus | P31942 | 1 | 107 | 346 | 31% | 4 | 11, 6 |
| Nucleolus | Q14562 | 2 | 375 | 1220 | 31% | 11 | 28, 9 |
| Nucleolus | Q9NQV6 | 3 | 352 | 1147 | 31% | 17 | 69, 6 |
| Nucleolus | Q14103 | 1 | 106 | 355 | 30% | 6 | 45, 6 |
| Nucleolus | Q8WTT2 | 1 | 240 | 800 | 30% | 6 | 42, 7 |
| Nucleolus | Q9BQ39 | 3 | 432 | 1474 | 29% | 8 | 17, 7 |
| Nucleolus | O15213 | 1 | 167 | 610 | 27% | 7 | 29, 8 |
| Nucleolus | Q68CQ4 | 1 | 202 | 756 | 27% | 6 | 45, 10 |
| Nucleolus | Q9NVP1 | 1 | 176 | 670 | 26% | 5 | 35, 7 |
| Nucleolus | O60832 | 1 | 132 | 514 | 26% | 5 | 18, 6 |
| Nucleolus | Q9BVP2 | 1 | 135 | 549 | 25% | 5 | 40, 17 |
| Nucleolus | P11142 | 1 | 155 | 646 | 24% | 6 | 27, 8 |
| Nucleolus | P21860 | 1 | 328 | 1342 | 24% | 11 | 39, 9 |
| Nucleolus | Q03701 | 1 | 239 | 1054 | 23% | 11 | 26, 7 |
| Nucleolus | Q12849 | 1 | 109 | 480 | 23% | 2 | 7, 6 |
| Nucleolus | Q99575 | 2 | 232 | 1024 | 23% | 11 | 58, 7 |
| Nucleolus | Q9NTJ3 | 2 | 296 | 1288 | 23% | 12 | 25, 6 |
| Nucleolus | Q9Y2K7 | 4 | 528 | 2324 | 23% | 6 | 34, 6 |
| Nucleolus | O00571 | 1 | 143 | 662 | 22% | 5 | 21, 7 |
| Nucleolus | P11021 | 1 | 141 | 654 | 22% | 5 | 15, 7 |
| Nucleolus | P49916 | 2 | 218 | 1009 | 22% | 9 | 20, 6 |
| Nucleolus | Q9UJV9 | 1 | 137 | 622 | 22% | 3 | 25, 18 |
| Nucleolus | Q13610 | 1 | 101 | 501 | 20% | 5 | 24, 6 |
| Nucleolus | Q5JTH9 | 1 | 264 | 1297 | 20% | 9 | 46, 6 |
| Nucleolus | Q8N122 | 2 | 204 | 1335 | 15% | 7 | 26, 7 |
| Nucleolus | Q9NS39 | 1 | 110 | 739 | 15% | 10 | 23, 7 |
| Nucleolus | O43143 | 1 | 111 | 795 | 14% | 4 | 30, 8 |
| Nucleolus | Q15003 | 1 | 101 | 741 | 14% | 15 | 29, 6 |
| Nucleolus | O75943 | 1 | 170 | 1362 | 12% | 3 | 48, 8 |
| Nucleolus | P55265 | 1 | 141 | 1226 | 12% | 19 | 35, 6 |
| Nucleolus | Q9Y6D6 | 2 | 227 | 1849 | 12% | 8 | 77, 9 |
| Nucleolus | Q08211 | 1 | 262 | 2540 | 10% | 6 | 24, 6 |
| Nucleolus | P78527 | 2 | 285 | 4128 | 7% | 7 | 12, 6 |
|  | | | | | | | |
| Sam68 body | Q07666 | 2 | 317 | 443 | 72% | 9 | 129, 6 |
|  | | | | | | | |
| Perinucleolar compartment | P31943 | 2 | 220 | 449 | 52% | 2 | 6, 7 |
|  | | | | | | | |
| PML Body | P38398 | 8 | 2420 | 3726 | 65% | 44 | 33, 6 |
| PML Body | Q92878 | 4 | 1534 | 2624 | 58% | 12 | 14, 6 |
| PML Body | P54132 | 5 | 1274 | 2834 | 45% | 16 | 26, 6 |
| PML Body | Q9NYB0 | 1 | 151 | 399 | 38% | 7 | 48, 8 |
| PML Body | P29590 | 5 | 668 | 1764 | 38% | 7 | 37, 6 |
| PML Body | P49959 | 1 | 252 | 708 | 36% | 6 | 33, 6 |
| PML Body | Q99638 | 1 | 128 | 391 | 33% | 3 | 35, 11 |
| PML Body | P43351 | 1 | 129 | 418 | 31% | 7 | 46, 10 |
| PML Body | Q13263 | 2 | 226 | 1670 | 14% | 12 | 53, 6 |
| PML Body | O75943 | 1 | 170 | 1362 | 12% | 3 | 48, 8 |
|  | | | | | | | |
| RNA pol II transcription | Q9HD15 | 1 | 109 | 236 | 46% | 4 | 39, 11 |
| RNA pol II transcription | Q9H9Y2 | 1 | 130 | 349 | 37% | 5 | 31, 6 |
| RNA pol II transcription | Q9BQ39 | 3 | 432 | 1474 | 29% | 8 | 17, 7 |
| RNA pol II transcription | P24928 | 1 | 479 | 1970 | 24% | 5 | 18, 10 |
| RNA pol II transcription | P17844 | 2 | 278 | 1842 | 15% | 5 | 13, 6 |
| RNA pol II transcription | Q08211 | 1 | 262 | 2540 | 10% | 6 | 24, 6 |
|  | | | | | | | |
| OPT domain | P14859 | 5 | 654 | 743 | 88% | 9 | 47, 7 |
| OPT domain | P08047 | 5 | 565 | 785 | 72% | 14 | 30, 6 |
|  | | | | | | | |
| PcG body | Q06587 | 2 | 264 | 406 | 65% | 6 | 66, 7 |
| PcG body | P35226 | 2 | 163 | 326 | 50% | 5 | 23, 9 |
| PcG body | Q99496 | 1 | 181 | 336 | 54% | 5 | 30, 7 |
| PcG body | Q9HC52 | 1 | 272 | 389 | 70% | 5 | 68, 11 |
| PcG body | O00257 | 1 | 487 | 560 | 87% | 13 | 46, 8 |
| PcG body | O95931 | 1 | 200 | 251 | 80% | 6 | 36, 9 |
| PcG body | Q13618 | 2 | 246 | 768 | 32% | 5 | 20, 7 |
| PcG body | Q15269 | 2 | 160 | 919 | 17% | 1 | 7 |
